# Comparison of 8-year knee osteoarthritis progression in 2 siblings: a case-based review

**DOI:** 10.1007/s10067-020-05181-6

**Published:** 2020-05-26

**Authors:** Margaret L. Gourlay, Linda L. Gourlay

**Affiliations:** 1 Boston Scientific Corporation, Marlborough, MA USA; 2 Department of Family Medicine, University of North Carolina, Chapel Hill, Manning Drive, CB #7595, Chapel Hill, NC 27599-7595 USA; 3 College of Nursing, University of Massachusetts, Amherst, MA USA; 4 Department of Psychiatry, Baystate Medical Center, Springfield, MA USA

**Keywords:** Cartilage, articular, Diagnostic imaging, Disease progression, Osteoarthritis, knee/physiopathology

## Abstract

Because the pathophysiology of knee osteoarthritis is poorly understood, optimal evidence-based clinical management is uncertain. Sibling comparison studies can help inform a clinical model to guide preventive care. We compared the 8-year clinical outcomes in 2 sisters with a family history of osteoarthritis, normal BMI, and absence of knee pain at baseline. Both patients had Kellgren–Lawrence grade 1 in the affected knee at the time of twisting knee injuries leading to osteoarthritis diagnoses at age 50 (patient 1) and 51 (patient 2). Patient 1 developed a chronic right knee effusion, and progressed to Kellgren–Lawrence grade 3 bilaterally by the time she had a right total knee replacement at age 58. Patient 2 had subchondral fractures of the right knee with transient effusion, which healed after 1 year of partial weight-bearing with crutches and subsequent daily use of knee sleeves. Patient 2 had Kellgren–Lawrence grade 0 bilaterally upon surveillance imaging at age 58. The terms “osteoarthritis and knee and diagnostic imaging and subchondral bone and pathophysiology” were searched in the PubMed database to identify original research articles to inform a clinical model consistent with the patients’ outcomes. A fluid model of osteoarthritis was the best explanatory model for the discordant clinical trajectories of the age-matched siblings. Patient recommendations are presented based on these findings.

**Electronic supplementary material:**

The online version of this article (10.1007/s10067-020-05181-6) contains supplementary material, which is available to authorized users.

## Introduction

Patients with knee osteoarthritis (OA) are treated conservatively until disease severity warrants joint replacement. While evidence evolves regarding OA pathophysiology and an optimal treatment regimen, sibling comparison studies can provide useful historical and practical clinical information for patients and clinicians. This report documents the medical history of knee OA in 2 sisters with divergent clinical courses. A literature search was conducted to explain the patients’ knee OA progression in the context of current data and to inform a clinical model to guide recommendations for patients.

## Case presentations

### Family history

The patients’ father (age 91 years, an only child) has obesity, hypertension, chronic kidney disease, and bilateral knee osteoarthritis with a history of bilateral knee replacements in his 80s. The patients’ paternal grandfather rubbed salve on his knees for pain relief, and their paternal grandmother had a postpartum exacerbation of hand arthritis in her early 30s for which she saw a subspecialist physician. The patients’ mother (age 91) has normal body weight, osteoporosis, and osteoarthritis and is wheelchair-bound after 2 strokes.

### Patient 1: 59-year-old woman with rapidly progressive bilateral knee OA

#### History

Patient 1 is a dual-certified psychiatric–mental health nurse practitioner and family nurse practitioner who has worked in academic medical and psychiatric clinical practice for 33 years. She had one child at 39 years of age. Before menopause, she participated in activities such as hiking, exercises with weights, and wearing fashion boots with 1-inch heels without pain. She has lived in 2-story houses with daily stairclimbing for a cumulative 38 years.

**Fig. 1:**
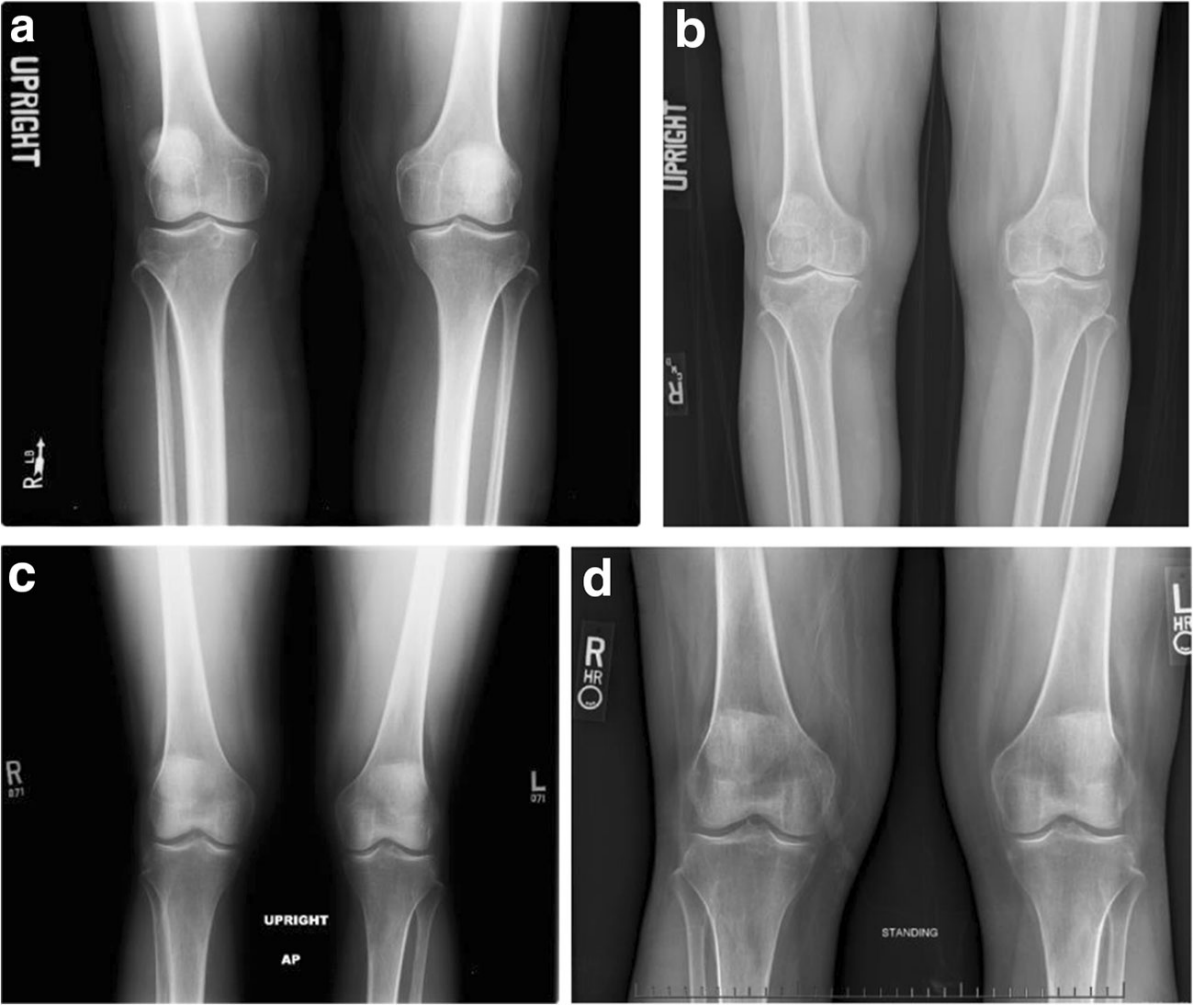
Bilateral anterior–posterior knee radiographs for patient 1 at age 50 (**a**) and age 58 (**b**), and for patient 2 at age 51 (**c**) [© 2015 BMJ Publishing Group Ltd. All rights reserved.] and age 58 (**d**)

Patient 1’s onset of chronic knee pain began during perimenopause at age 50. She was seen by an orthopedic surgeon because of pain and an intermittent sharp catching sensation in her anterior left knee. Physical examination findings included height 5′ 5″, weight 136 pounds (BMI 22.6), left knee range of motion 0–130° with crepitus throughout femoral joint, 1+ effusion, and medial joint line tenderness. Radiographs showed the left knee with mild narrowing of the medial joint space compared with the right knee and slight malalignment of the patellofemoral joint (Fig. 1a). She was diagnosed with patellofemoral osteoarthritis and medial meniscus tears of the left knee later confirmed on magnetic resonance imaging (MRI).

Over the subsequent 2 years, patient 1 had 2 arthroscopic examinations with cartilage trimming—first of the left knee and later of the right knee after she tripped and fell onto a mat. At age 52, patient 1 developed a chronic right knee effusion after she stepped off a bus and her knee twisted and buckled during touchdown in a gravel parking lot. Her bilateral knee pain steadily progressed during subsequent years, and she noted increasing varus deformity of both knees. By age 58, radiographs showed definite narrowing of the right and left knee joint spaces (Kellgren–Lawrence grade 3; Fig. 1b). Patient 1’s knee pain was worse in the right knee. She had exacerbation of pain when weight-bearing and increased pain and a sensation of pressure during prolonged sitting, making her unable to tolerate long car rides.

#### Treatment

Patient 1 received various treatments including nonsteroidal anti-inflammatory agents, topical lidocaine patches, corticosteroid injections, viscosupplementation, knee braces (offloader and sleeve braces), and physical therapy. Based on her worsening pain and increasing frequency of near falls, her orthopedist recommended bilateral total knee replacement. She underwent a right total knee replacement at age 58. After in-home physical therapy for 4 weeks, she attended weekly outpatient physical therapy appointments for 2 months. She then returned to clinical practice and reports good healing of the right knee (pain-free) and persistent left knee pain that is continuous during weight-bearing and resolves when nonweight-bearing. She hopes to defer a left total knee replacement as long as possible.

At age 50, patient 1 was diagnosed with mild vitamin D deficiency (serum 25-OH vitamin D 15.0 ng/ml). After 3 months of supplementation, she was vitamin D replete (serum 25-OH vitamin D 44.5 ng/ml). She eventually stopped taking supplements and was not tested again until age 59, when she was found to be severely vitamin D–deficient (serum 25-OH vitamin D 8.2 ng/ml) during a workup for bone density loss in her mandible. A dual-energy x-ray absorptiometry (DXA) bone density test revealed osteopenia, with the lowest *T* score − 2.3 at the femoral neck.

### Patient 2: 58-year-old woman with mild knee OA and hand OA

#### History

Patient 2 is a family physician who practiced outpatient and inpatient primary care medicine for 15 years. She has a history of pes planus, valgus deformity of the knees for which she consciously torqued her ankles outward to normalize knee alignment since childhood, and osteoporosis diagnosed in her late 30s (lowest *T* score − 3.0 at the lumbar spine). She has lived in ground-level housing for a cumulative 55 years (3 years in apartments or houses with stairs). Between ages 40 and 50, she enjoyed walking 4 miles per day, several times per week.

At age 51 (7 months after her last menstrual period), patient 2 had new onset of knee pain after very prolonged standing and a twisting injury to her right knee while exiting an elevator during an 18-h hospital shift. Physical examination 2 months later showed height 5′ 7″, weight 125 pounds (BMI 19.5), and tenderness to palpation at the right knee lateral joint line and femoral condyle. Radiographs showed a small right knee effusion and minimal osteophytosis with no joint space narrowing (Kellgren–Lawrence grade 1 (right) and grade 0 (left); Fig. 1c), while MRI revealed 2 small subchondral insufficiency fractures at the right lateral femoral condyle with a large amount of associated marrow edema, and a complex, predominantly horizontal tear involving the posterior horn of the medial meniscus. Immediately after the fractures, she felt constant right knee pain (while weight-bearing or nonweight-bearing) with increased pain upon weight-bearing, and a sensation of increasing pressure and pain while sitting with her right leg in a dependent position. She was treated with partial weight-bearing on crutches until 14 months after the injury, viscosupplementation at 4 months, and teriparatide treatment to improve bone healing at 7 months. Her clinical course through 2015 is published separately as a case report [1].

Since 2015, patient 2 has walked independently with the use of bilateral elasticized knee sleeves at all times outside her home and most of the time within the home. Although physical therapists discouraged the use of knee sleeves because they might promote muscle weakness, patient 2 considered published evidence [2–5] and reasoned that wearing the sleeves would increase her muscle strength long-term by enabling her to walk more often. When walking without knee sleeves, she experiences numbness, transient sharp pains and a sensation of walking on an unhealed wound, especially at the right posterolateral tibial plateau where the subchondral fractures occurred. She has minimal knee pain while sleeping on her back but aching and numbness when her knee bones shift as she lies on either side. At age 56, she moved out of state for caregiving responsibilities lasting 6 months. At age 57, she began full-time work as a medical writer for industry, having full control of periods of sitting and standing.

Also since 2015, patient 2 experienced sharp, reversible pain in the distal interphalangeal (DIP) and proximal interphalangeal (PIP) joints during prolonged periods of typing, sometimes followed by soreness that persists for hours after typing. However, prolonged periods of rest from typing during holidays, and sleeping on her side with her head resting on her hands, also led to hand puffiness and pain. At age 58, she consulted her primary care physician, who ordered serology and radiographs. Serology revealed positive ANA and anti-centromere B antibody tests, hand radiographs showed periarticular lucencies bilaterally without bony erosions, and surveillance knee radiographs showed preserved joint spaces (Kellgren–Lawrence grade 0 bilaterally; Fig. 1d). A referral rheumatologist documented her clinical diagnosis as inconsistent with inflammatory osteoarthritis, concluding the ANA result was false-positive.

#### Treatment

In addition to daily use of knee sleeves bilaterally, patient 2 uses cold packs on her knees, diclofenac 1% topical (especially for hands) daily, and oral magnesium salicylate or meloxicam 15 mg as needed, usually less than once daily. She uses a gaming computer keyboard with cherry-red MX keys (lowest level of resistance) for all typing and voice dictation software for longer documents. She wears crafter’s gloves while typing, sometimes using painter’s tape to stabilize the PIP and DIP joints.

### Comparison of clinical trajectories

Characteristics of patient 1 and patient 2 are summarized in Table 1. Differences include a higher BMI, longer period of knee effusion, much longer cumulative daily stairclimbing, vitamin D deficiency, much less use of knee sleeves, and absence of hand pain in patient 1 compared with patient 2. Patient 1’s knee radiographs evolved from Kellgren–Lawrence grade 1 (right) and grade 0 (left) (Fig. 1a) to grade 3 bilaterally (Fig. 1b) by 8 years post-injury, while patient 2’s knee radiographs showed Kellgren–Lawrence grade 1 (right) and grade 0 (left) at the time of her right subchondral fractures (Fig. 1c) and grade 0 bilaterally at 7 years post-injury (Fig. 1d). In both patients, symptomatology correlated with imaging markers of fluid (effusion and bone marrow edema) and structural damage in joint tissue, primarily medial in patient 1 and lateral in patient 2.

**Table 1:** Characteristics of patient 1 (rapidly progressive knee OA) and patient 2 (stable knee OA)

Characteristic	Patient 1	Patient 2
BMI at age 50–51 years	22.6	19.5
BMI at age 58	24.8	20.2
Knee joint alignment	Varus	Valgus
Parity	1 child	Nulliparous
Twisting knee injuries	2	1
Knee effusion (cumulative years)	8	< 1
Hand pain	No	Yes
Daily stairclimbing (cumulative years)	38	< 1
Daily knee sleeve use in affected knee (cumulative years)	< 1 (used brace instead)	7
Kellgren–Lawrence grades at age 50–51 years	1 (right), 0 (left)	1 (right), 0 (left)
Kellgren–Lawrence grades at age 58 years	3 bilaterally	0 bilaterally
Primary location of imaging abnormalities	medial	lateral
Serum 25-OH vitamin D (ng/ml)	15.0 at age 50; 44.5 at age 50 (after prescription vitamin D supplements); 8.2 at age 59	24.0 at age 50; 53.1 at age 56
Lowest DXA bone density *T* score	− 2.3 at age 59	− 3.0 at age 52

*DXA* dual-energy x-ray absorptiometry

## Literature search

**Fig. 2:**
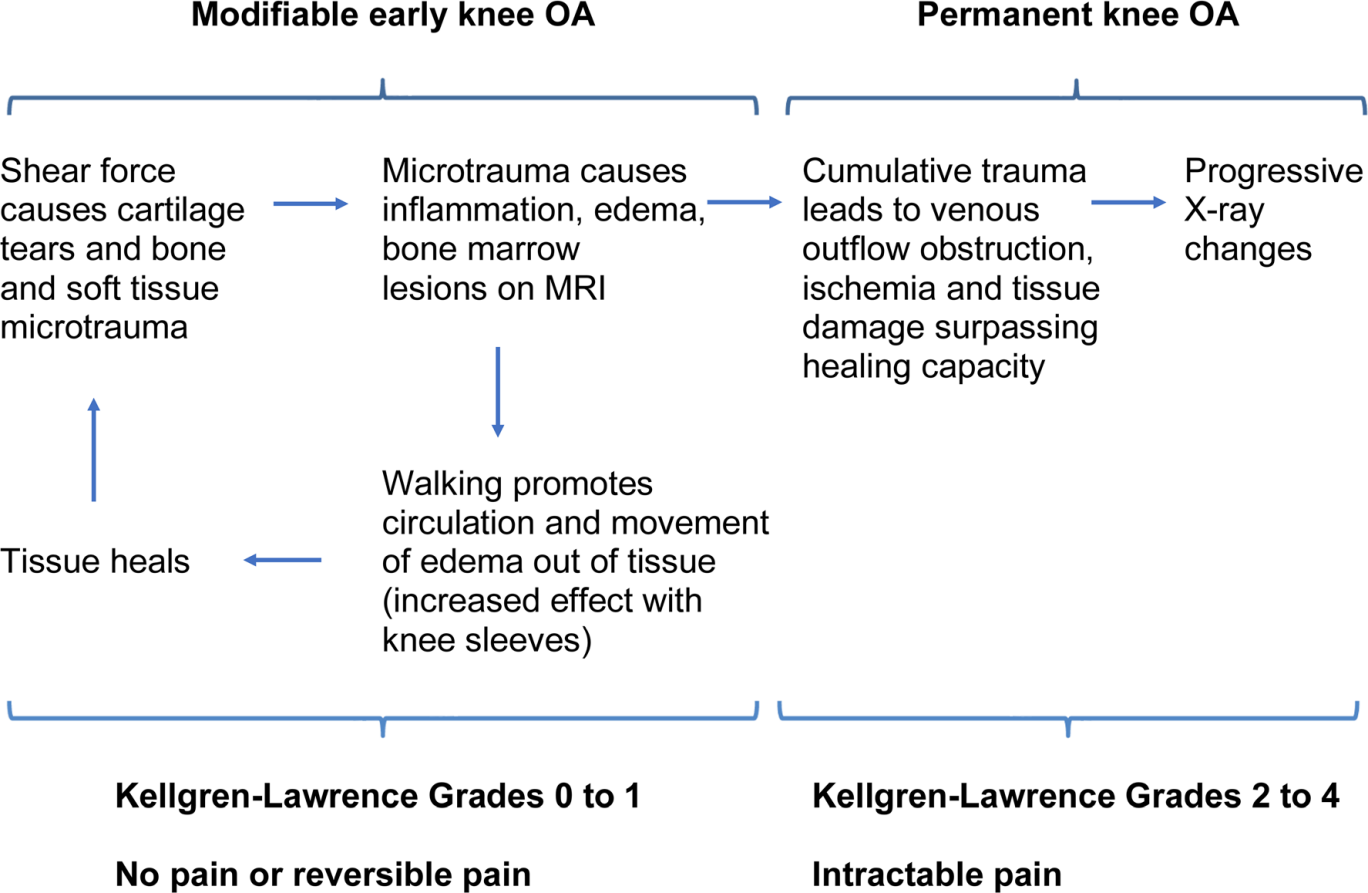
Proposed clinical model of knee osteoarthritis progression

The PubMed/MEDLINE database was searched using the Boolean search phrase “osteoarthritis and knee and diagnostic imaging and subchondral bone and pathophysiology” to identify original research articles published from inception to March 13, 2020. These search terms were selected for their relevance to a pathophysiological model for knee osteoarthritis progression including subchondral bone (because of the reversibility/healing capacity suggested by patient 2’s clinical course) and imaging markers. The search yielded 135 articles (Supplementary Appendix) of which 113 were excluded by title, abstract, or full-text review for the following reasons: study of a medical treatment, surgical study, or procedure (23), bone density or mechanical properties of bone or cartilage (19), specific patient or animal subgroup (12), posttraumatic osteoarthritis (6), or receptors, hormones, or biological markers (6); interventional study (14); test of imaging technique (7); histological or morphometric study (5); review article (14) or letter (1); non-English language (6). Twenty-two articles were eligible, of which 13 [6–18] were cited because they had the highest relevance to inform the proposed clinical model (Fig. 2). Supplemental searches were performed to expand on key topics identified in the original literature search, i.e., subchondral bone pathology [1], knee sleeves [2–5, 19–21], bone marrow lesions [22–26], clinical risk factors for knee OA progression [27–33], OA phenotypes [34], nonpharmacological treatments for OA [35–43], and clinical practice guidelines for OA management [44].

## Discussion

### Proposed clinical model

The authors’ clinical trajectories are consistent with Lee et al.’s fluid model of osteoarthritis [6] and Aaron et al.’s report of subchondral bone circulation in knee OA [7], both based on dynamic contrast-enhanced MRI findings. As an extension of these models, our clinical model of knee osteoarthritis progression (Fig. 2) involves all fluid (including blood flow, edema, and effusion) and tissues within the knee joint and includes a modifiable early phase (Kellgren–Lawrence grades 0–1) in which the subchondral bone and soft tissue are capable of healing if excess fluid can be shifted out of tissue by walking. After repeated reinjury, patients transition to a nonmodifiable stage of knee OA where permanent structural damage can no longer be healed by driving out excess fluid. This permanent stage is radiographically detectable as Kellgren–Lawrence grades 2–4.

Consistent with the fluid-based clinical model, both patients experienced a feeling of fullness and pressure while they had knee joint effusions. Immediately post-injury, pain increased when the injured leg was in a dependent position and with use of a knee sleeve, presumably due to ischemic pain from fluid compression. This feeling of pressure persisted for patient 1 (who had a chronic effusion) and resolved for patient 2 (whose effusion resolved). Compared with nonweight-bearing, patient 2’s partial weight-bearing during healing of her subchondral fractures might have driven more fluid out of the knee joint tissues, allowing successful healing of the fractures. Later, knee sleeves might have accentuated the squeezing action of leg muscles, allowing more rapid fluid clearance as patient 2’s edema regressed. In contrast, patient 1’s chronic effusion surpassed the capacity of walking to move fluid out of her knee joint tissues, promoting prolonged increased pressure within the knee joint space (similar to compartment syndrome) that led to ischemia, cell death, and permanent structural damage.

Patient 2’s finger pain upon overuse of fingers (promoting mild inflammation) and upon prolonged hand rest or compression of fingers during sleep (promoting edema) is also consistent with transient OA pain associated with fluid shifts in joint tissues.

### Comparison with other studies

Bone marrow lesions (sometimes called bone marrow edema) have been demonstrated to be present in individuals without [8, 22] and with [23] clinical OA; to wax and wane with weight-bearing activity (e.g., in marathon runners [24, 25]); and to be associated with incident knee pain in individuals at risk of OA [26], and with pain and disability for activities of daily living in patients with established OA [9]. Joint fluid overload correlates with symptoms. For example, Kornaat et al. reported that large joint effusion was associated with pain (OR, 9.99; 99% CI 1.28, 149) and stiffness (OR, 4.67; 99% CI 1.26, 26.1), and the presence of an osteophyte in the patellofemoral compartment (OR, 2.25; 99% CI 1.06, 4.77) was associated with pain in patients with osteoarthritis of the knee; all other imaging findings (including bone marrow edema) were not associated with symptoms in their study [10].

Budzik et al. found that subchondral bone marrow (SCBM) vascularization was greatest in the compartment most affected by OA, both in tibia and femur, with a positive correlation between the extent of bone marrow edema and SCBM perfusion [11]. Lee et al. hypothesized that changes in perfusion patterns in subchondral bone bear a functional relationship to bone remodeling and cartilage degeneration [6]. Both Lee and Aaron emphasized venous outflow obstruction as a key mechanism of disease, where venous stasis can affect intraosseous pressure, can reduce arterial inflow, can reduce oxygen content, and may contribute to altered cell signaling in the pathophysiology of OA [7]. As described above, we incorporated these fluid dynamics as the core principles in our clinical model.

In an integrated joint system model of OA, Edd et al. described close interrelationships among cartilage thickness, gait mechanics, and subchondral bone [12]. Like their model, our model includes an early and late stage of OA during which the strength of these interrelationships may change with disease progression.

Collectively, these findings suggest a normal pattern of fluid shifts in response to low-grade inflammation associated with weight-bearing activity and injury. When an inciting injury causes a meniscal tear, inflammation, edema, and fluid shifts within joint tissue are usually initially reversible, and tissue can heal. If fluid overload is severe and sustained (as in patient 1), fluid compressive forces promote an ischemic state which leads to worsening irreparable structural damage, including subchondral bone attrition [13] and denudation [14] or osteonecrosis with rapid OA progression [15]. With such a model, some previously studied patient phenotypes (e.g., accelerated knee OA [16], inflammatory or bone phenotypes [34]) may represent different stages of the same pathophysiological process, with disease progression rate determined by risk factors such as female gender (e.g., different lower leg joint morphometry compared with men) [17, 27], body weight [28], joint malalignment [13, 18], family history (genetic tendency toward frail cartilage, obesity, or joint malalignment) [29], and possibly vitamin D deficiency [30, 31] (as for patient 1).

### Importance of nonpharmacological treatments

Nonpharmacological treatments are a mainstay of OA management and can potentially help slow the progression of knee OA [35]. The most important nonpharmacological treatments used by the patients in the case studies are discussed.

#### Lifestyle modification (weight reduction, diet, and exercise)

An 18-month randomized controlled trial (RCT) of 454 overweight and obese adults with knee OA found a significant dose–response to weight loss; participants who lost > 10% of baseline body weight had significantly (*p* = 0.0001) lower resultant knee forces and lower leg muscle forces than participants with less weight loss [36], and participants in diet-plus-exercise and diet-only groups had more weight loss and greater reductions in IL-6 levels than those in an exercise-only group [37]. The benefits of exercise may decrease as OA severity progresses to an advanced and irreversible stage. For example, a prospective study of 1059 patients with radiographic knee OA at baseline found that in those with advanced disease (Kellgren–Lawrence grade 4), greater daily minutes in physical activity were associated with worsening symptoms [42].

#### Cryotherapy and heat

Patients may prefer heat, cold, or contrasting temperatures to alleviate OA pain [38, 39]. According to our proposed clinical model, cryotherapy might be expected to alleviate pain by temporarily reducing blood flow to joint tissues. A systematic review of RCTs identified insufficient primary studies to draw conclusions about the effectiveness of cryotherapy on pain and physical function on individuals with knee osteoarthritis [40]. However, a review of cryotherapy treatment after unicompartmental and total knee arthroplasty concluded that continuous circulating cold flow might be optimal for pain relief and minimization of pain medication use after knee arthroplasty [41]. These findings suggest that cold therapy may be especially helpful for OA patients during periods of increased inflammation (e.g., after surgery).

#### Knee sleeves and braces

In patients without knee effusion, a snugly fitting elasticized knee sleeve can improve knee biomechanics [2, 19], enable surefooted walking, and minimize lower extremity edema via mild compression (below the threshold for ischemia). Consistent with our findings, knee sleeve use is associated with improved activity scores and pain scores for patients with lower Kellgren–Lawrence grades (as for patient 2) [20], while corrective or realignment braces are more effective in moderate or severe OA (patient 1) [21]. While patient 2 maintained stable Kellgren–Lawrence grade 0–1 during a 7-year period of knee sleeve use, the association between knee sleeve use and rate of radiographic OA progression has not been tested in an RCT.

## Conclusions and recommendations to patients

The focus of knee osteoarthritis preventive care should be minimization of fluid accumulation in the knee joint to prevent ischemic tissue compromise that eventually leads to permanent structural damage. The broad categories of no pain versus reversible pain (resolves during nonweight-bearing) versus intractable pain are clinically useful because they correlate with smaller to larger degrees of fluid overload and imaging findings.

Based on the reported findings and the proposed clinical model, we recommend the preventive measures below for patients. These recommendations are consistent with the 2019 American College of Rheumatology/Arthritis Foundation Guideline for the Management of Osteoarthritis of the Hand, Hip, and Knee [44] and offer additional strategies for prevention and pain management based on the proposed fluid model of OA:

1. Lifestyle habits should be optimized to attain and maintain normal body weight [28].
2. Daily walking (ideally ≥ 6000 steps/day) as tolerated is encouraged because it is associated with less risk of functional limitation over 2 years [43].
3. Torsion (twisting) of the knee during weight-bearing, and pushing against resistance (e.g., pushing a heavy vacuum cleaner) or while carrying a weighted load must be avoided because these activities apply shear force to the knee that can tear cartilage [33], especially in patients with genetic tendencies toward weak cartilage.
4. Prolonged standing must be avoided because it increases lower extremity edema and (if extreme) increases the risk of subchondral fractures [1]. Patients should stand on thick rugs and commercial grade cushioned floor mats to minimize knee joint strain.
5. While stairclimbing might benefit the knee by building thigh muscle strength, frequent knee-bending activities (including stairclimbing) are associated with a higher prevalence of knee cartilage lesions and increased risk of progression of cartilage and meniscal lesions in asymptomatic middle-aged subjects [32]. Stairclimbing may be one reason patient 1 had more rapid disease progression than patient 2.
6. Knee sleeves are a safe and effective nonpharmacological intervention for patients with early-stage knee OA [2–5, 19, 20]. However, patients with worsening pain upon use of a knee sleeve should stop use and consult a physician to rule out knee joint effusion.
7. Consistent with our extension of Aaron’s [7] and Lee’s [6] hypotheses regarding perfusion kinetics, vasoactive properties can be exploited for short-term knee pain relief via vasoconstriction. Cold packs should be used preferentially to minimize long-term side effects from pharmacological treatments. Patients can direct cold water onto their knees while waiting for water to warm up in the shower, and knees can be left uncovered during sleep in a cool room to decrease pain at night.
8. Hand exercise is strongly recommended for patients with hand OA [44]. Consistent with our clinical model, patients should aim for an optimal amount of hand exercise to drive excess fluid out of the fingers while avoiding overuse that can lead to inflammation. To decrease repetitive strain on fingers, the authors have used voice dictation software, a light-touch computer keyboard, plumbing fixtures with bar handles, and jars with pump dispensers that can be operated using the palm, and the handle of an eating utensil to break the seal on a jar before opening. Cold therapy should only be used for short periods on hands to avoid reduced circulation in end-organ blood vessels of the fingers.
9. Patients should consult their primary care provider if they develop intractable joint pain or a lower threshold of weight-bearing activity leading to pain, since chronic pain irreversibility may suggest permanent structural joint tissue damage.

## Electronic supplementary material

ESM 1 (DOCX 86 kb).
