# Correction to: Comparison of 8-year knee osteoarthritis progression in 2 siblings: a case-based review

**DOI:** 10.1007/s10067-020-05278-y

**Published:** 2020-07-15

**Authors:** Margaret L. Gourlay, Linda L. Gourlay

**Affiliations:** 1 Boston Scientific Corporation, Marlborough, MA USA; 2 Department of Family Medicine, University of North Carolina, Chapel Hill, Manning Drive, CB #7595, Chapel Hill, NC 27599-7595 USA; 3 College of Nursing, University of Massachusetts, Amherst, MA USA; 4 Department of Psychiatry, Baystate Medical Center, Springfield, MA USA

**Correction to: Clinical Rheumatology**

10.1007/s10067-020-05181-6

The article “Comparison of 8-year knee osteoarthritis progression in 2 siblings: a case-based review”, written by Margaret L. Gourlay and Linda L. Gourlay, was originally published electronically on the publisher’s internet portal on 26 May 2020 without open access. With the author(s)’ decision to opt for Open Choice the copyright of the article changed on 15 July 2020 to © The Author(s) 2020 and the article is forthwith distributed under a Creative Commons Attribution 4.0 International License (https://creativecommons.org/licenses/by/4.0/), which permits use, sharing, adaptation, distribution and reproduction in any medium or format, as long as you give appropriate credit to the original author(s) and the source, provide a link to the Creative Commons license, and indicate if changes were made.

The author also wanted to note that in Figure 1c legend the copyright “© 2015 BMJ Publishing Group Ltd. All rights reserved.” should have been added. The original article has been corrected.

