# Supplementary material for: Comparison of 8-year knee osteoarthritis progression in 2 siblings: a case-based review
Source: Clin Rheumatol. 2020 May 26;39(10):3105–13. doi: 10.1007/s10067-020-05181-6 (PMC7497332; doi:10.1007/s10067-020-05181-6)
Supplement: Supplementary file 1 — (DOCX 86 kb). [file 10067_2020_5181_MOESM1_ESM.docx]

**Supplementary Appendix**

Supplement to: Gourlay ML, Gourlay LL. Comparison of 8-year knee osteoarthritis progression in 2 siblings: a case-based review

**PubMed search of "osteoarthritis and knee and diagnostic imaging and subchondral bone and pathophysiology" on March 13, 2020**

**ELIGIBILITY STATUS OF ARTICLES (135)**

Inclusions (22)

**Bright green**: eligible (22)

Exclusions (113)

**Blue:** study of a medical treatment, surgical study or procedure (23)

Orange letters: study of bone density or mechanical properties of bone (19)

**Yellow:** specific patient or animal subgroup (12)

Red: posttraumatic osteoarthritis (6)

**Light green**: study of receptors, hormones or biological markers (6)

Orange: interventional study (14)

Green letters: test of new imaging technique (7)

Brown: histological or morphometric study (5)

Grey: review article or letter (15)

Red letters: non-English language (6)

| 1. | [Reduced bone activity in the native compartments after medial mobile-bearing unicompartmental knee arthroplasty. A prospective SPECT/CT study.](https://www.ncbi.nlm.nih.gov/pubmed/31362550)  REJECT: surgical procedure |
| --- | --- |
|  | Beckers L, Ooms D, Berger P, Van Laere K, Scheys L, Vandenneucker H. |
|  | Bone Joint J. 2019 Aug;101-B(8):915-921. doi: 10.1302/0301-620X.101B8.BJJ-2018-1569.R1. |
|  | PMID: 31362550 [PubMed - indexed for MEDLINE] |
|  | [Similar articles](https://www.ncbi.nlm.nih.gov/pubmed?linkname=pubmed_pubmed&from_uid=31362550) |

| 2. | [Comparison of total knee arthroplasty after combined high tibial osteotomy with a matched group of primary total knee arthroplasty.](https://www.ncbi.nlm.nih.gov/pubmed/31291853)  REJECT: surgical procedure |
| --- | --- |
|  | Papp M, Zsákai Z, Gömöri A. |
|  | Eklem Hastalik Cerrahisi. 2019 Aug;30(2):79-84. doi: 10.5606/ehc.2019.66900. |
|  | PMID: 31291853 [PubMed - indexed for MEDLINE]**Free Article** |
|  | [Similar articles](https://www.ncbi.nlm.nih.gov/pubmed?linkname=pubmed_pubmed&from_uid=31291853) |

| 3. | [Knee osteoarthritis patients with more subchondral cysts have altered tibial subchondral bone mineral density.](https://www.ncbi.nlm.nih.gov/pubmed/30611224)  REJECT: patient subgroup--arthroplasty |
| --- | --- |
|  | Burnett WD, Kontulainen SA, McLennan CE, Hazel D, Talmo C, Wilson DR, Hunter DJ, Johnston JD. |
|  | BMC Musculoskelet Disord. 2019 Jan 5;20(1):14. doi: 10.1186/s12891-018-2388-9. |
|  | PMID: 30611224 [PubMed - indexed for MEDLINE]**Free PMC Article** |
|  | [Similar articles](https://www.ncbi.nlm.nih.gov/pubmed?linkname=pubmed_pubmed&from_uid=30611224) |

| 4. | [Accelerated Knee Osteoarthritis Is Characterized by Destabilizing Meniscal Tears and Preradiographic Structural Disease Burden.](https://www.ncbi.nlm.nih.gov/pubmed/30592385) |
| --- | --- |
|  | Driban JB, Davis JE, Lu B, Price LL, Ward RJ, MacKay JW, Eaton CB, Lo GH, Barbe MF, Zhang M, Pang J, Stout AC, Harkey MS, McAlindon TE. |
|  | Arthritis Rheumatol. 2019 Jul;71(7):1089-1100. doi: 10.1002/art.40826. Epub 2019 May 21. |
|  | PMID: 30592385 [PubMed - indexed for MEDLINE] |
|  | [Similar articles](https://www.ncbi.nlm.nih.gov/pubmed?linkname=pubmed_pubmed&from_uid=30592385) |

| 5. | [Tougu Xiaotong capsule exerts a therapeutic effect on knee osteoarthritis by regulating subchondral bone remodeling.](https://www.ncbi.nlm.nih.gov/pubmed/30592265)  REJECT: medical treatment |
| --- | --- |
|  | Wu G, Zhang J, Chen W, Chen S, Huang Y, Lin R, Huang M, Li Z, Zheng L, Li X. |
|  | Mol Med Rep. 2019 Mar;19(3):1858-1866. doi: 10.3892/mmr.2018.9778. Epub 2018 Dec 20. |
|  | PMID: 30592265 [PubMed - indexed for MEDLINE] |
|  | [Similar articles](https://www.ncbi.nlm.nih.gov/pubmed?linkname=pubmed_pubmed&from_uid=30592265) |

| 6. | [Use of a Biomimetic Scaffold for the Treatment of Osteochondral Lesions in Early Osteoarthritis.](https://www.ncbi.nlm.nih.gov/pubmed/30515412)  REJECT: medical treatment |
| --- | --- |
|  | Condello V, Filardo G, Madonna V, Andriolo L, Screpis D, Bonomo M, Zappia M, Dei Giudici L, Zorzi C. |
|  | Biomed Res Int. 2018 Nov 1;2018:7937089. doi: 10.1155/2018/7937089. eCollection 2018. |
|  | PMID: 30515412 [PubMed - indexed for MEDLINE]**Free PMC Article** |
|  | [Similar articles](https://www.ncbi.nlm.nih.gov/pubmed?linkname=pubmed_pubmed&from_uid=30515412) |

| 7. | [Deficiency of the pattern-recognition receptor CD14 protects against joint pathology and functional decline in a murine model of osteoarthritis.](https://www.ncbi.nlm.nih.gov/pubmed/30485272)  REJECT: study of receptors, hormones or biological markers |
| --- | --- |
|  | Sambamurthy N, Zhou C, Nguyen V, Smalley R, Hankenson KD, Dodge GR, Scanzello CR. |
|  | PLoS One. 2018 Nov 28;13(11):e0206217. doi: 10.1371/journal.pone.0206217. eCollection 2018. |
|  | PMID: 30485272 [PubMed - indexed for MEDLINE]**Free PMC Article** |
|  | [Similar articles](https://www.ncbi.nlm.nih.gov/pubmed?linkname=pubmed_pubmed&from_uid=30485272) |

| 8. | [Bone marrow lesion is associated with disability for activities of daily living in patients with early stage knee osteoarthritis.](https://www.ncbi.nlm.nih.gov/pubmed/30187274) |
| --- | --- |
|  | Sadatsuki R, Ishijima M, Kaneko H, Liu L, Futami I, Hada S, Kinoshita M, Kubota M, Aoki T, Takazawa Y, Ikeda H, Okada Y, Kaneko K. |
|  | J Bone Miner Metab. 2019 May;37(3):529-536. doi: 10.1007/s00774-018-0950-z. Epub 2018 Sep 5. |
|  | PMID: 30187274 [PubMed - indexed for MEDLINE] |
|  | [Similar articles](https://www.ncbi.nlm.nih.gov/pubmed?linkname=pubmed_pubmed&from_uid=30187274) |

| 9. | [Magnoflorine with hyaluronic acid gel promotes subchondral bone regeneration and attenuates cartilage degeneration in early osteoarthritis.](https://www.ncbi.nlm.nih.gov/pubmed/30149068)  REJECT: medical treatment |
| --- | --- |
|  | Cai Z, Feng Y, Li C, Yang K, Sun T, Xu L, Chen Y, Yan CH, Lu WW, Chiu KY. |
|  | Bone. 2018 Nov;116:266-278. doi: 10.1016/j.bone.2018.08.015. Epub 2018 Aug 24. |
|  | PMID: 30149068 [PubMed - indexed for MEDLINE] |
|  | [Similar articles](https://www.ncbi.nlm.nih.gov/pubmed?linkname=pubmed_pubmed&from_uid=30149068) |

| 10. | [Modeling knee osteoarthritis pathophysiology using an integrated joint system (IJS): a systematic review of relationships among cartilage thickness, gait mechanics, and subchondral bone mineral density.](https://www.ncbi.nlm.nih.gov/pubmed/30056214) |
| --- | --- |
|  | Edd SN, Omoumi P, Andriacchi TP, Jolles BM, Favre J. |
|  | Osteoarthritis Cartilage. 2018 Nov;26(11):1425-1437. doi: 10.1016/j.joca.2018.06.017. Epub 2018 Jul 26. |
|  | PMID: 30056214 [PubMed - indexed for MEDLINE]**Free Article** |
|  | [Similar articles](https://www.ncbi.nlm.nih.gov/pubmed?linkname=pubmed_pubmed&from_uid=30056214) |

| 11. | [Subchondral bone circulation in osteoarthritis of the human knee.](https://www.ncbi.nlm.nih.gov/pubmed/29723635) |
| --- | --- |
|  | Aaron RK, Racine JR, Voisinet A, Evangelista P, Dyke JP. |
|  | Osteoarthritis Cartilage. 2018 Jul;26(7):940-944. doi: 10.1016/j.joca.2018.04.003. Epub 2018 May 1. |
|  | PMID: 29723635 [PubMed - indexed for MEDLINE]**Free Article** |
|  | [Similar articles](https://www.ncbi.nlm.nih.gov/pubmed?linkname=pubmed_pubmed&from_uid=29723635) |

| 12. | [The effect of ageing and osteoarthritis on the mechanical properties of cartilage and bone in the human knee joint.](https://www.ncbi.nlm.nih.gov/pubmed/29651151)  REJECT: study of bone density or mechanical properties of bone |
| --- | --- |
|  | Peters AE, Akhtar R, Comerford EJ, Bates KT. |
|  | Sci Rep. 2018 Apr 12;8(1):5931. doi: 10.1038/s41598-018-24258-6. |
|  | PMID: 29651151 [PubMed - indexed for MEDLINE]**Free PMC Article** |
|  | [Similar articles](https://www.ncbi.nlm.nih.gov/pubmed?linkname=pubmed_pubmed&from_uid=29651151) |

| 13. | [How Do MRI-Detected Subchondral Bone Marrow Lesions (BMLs) on Two Different MRI Sequences Correlate with Clinically Important Outcomes?](https://www.ncbi.nlm.nih.gov/pubmed/29441423)  REJECT: test of new imaging technique |
| --- | --- |
|  | Mattap SM, Aitken D, Wills K, Laslett L, Ding C, Pelletier JP, Martel-Pelletier J, Graves SE, Lorimer M, Cicuttini F, Jones G. |
|  | Calcif Tissue Int. 2018 Aug;103(2):131-143. doi: 10.1007/s00223-018-0402-8. Epub 2018 Feb 13. |
|  | PMID: 29441423 [PubMed - indexed for MEDLINE] |
|  | [Similar articles](https://www.ncbi.nlm.nih.gov/pubmed?linkname=pubmed_pubmed&from_uid=29441423) |

| 14. | [Relationships between in vivo dynamic knee joint loading, static alignment and tibial subchondral bone microarchitecture in end-stage knee osteoarthritis.](https://www.ncbi.nlm.nih.gov/pubmed/29382604)  REJECT: patient subgroup |
| --- | --- |
|  | Roberts BC, Solomon LB, Mercer G, Reynolds KJ, Thewlis D, Perilli E. |
|  | Osteoarthritis Cartilage. 2018 Apr;26(4):547-556. doi: 10.1016/j.joca.2018.01.014. Epub 2018 Jan 31. |
|  | PMID: 29382604 [PubMed - indexed for MEDLINE]**Free Article** |
|  | [Similar articles](https://www.ncbi.nlm.nih.gov/pubmed?linkname=pubmed_pubmed&from_uid=29382604) |

| 15. | [Bone marrow lesions in osteoarthritis: What lies beneath.](https://www.ncbi.nlm.nih.gov/pubmed/29266428)  REJECT: review |
| --- | --- |
|  | Alliston T, Hernandez CJ, Findlay DM, Felson DT, Kennedy OD. |
|  | J Orthop Res. 2018 Jul;36(7):1818-1825. doi: 10.1002/jor.23844. Epub 2018 May 22. Review. |
|  | PMID: 29266428 [PubMed - indexed for MEDLINE]**Free Article** |
|  | [Similar articles](https://www.ncbi.nlm.nih.gov/pubmed?linkname=pubmed_pubmed&from_uid=29266428) |

| 16. | [Subchondral bone fragility with meniscal tear accelerates and parathyroid hormone decelerates articular cartilage degeneration in rat osteoarthritis model.](https://www.ncbi.nlm.nih.gov/pubmed/29251375)  REJECT: study of receptors, hormones or biological markers |
| --- | --- |
|  | Morita Y, Ito H, Ishikawa M, Fujii T, Furu M, Azukizawa M, Okahata A, Tomizawa T, Kuriyama S, Nakamura S, Nishitani K, Yoshitomi H, Matsuda S. |
|  | J Orthop Res. 2018 Jul;36(7):1959-1968. doi: 10.1002/jor.23840. Epub 2018 Jan 16. |
|  | PMID: 29251375 [PubMed - indexed for MEDLINE]**Free Article** |
|  | [Similar articles](https://www.ncbi.nlm.nih.gov/pubmed?linkname=pubmed_pubmed&from_uid=29251375) |

| 17. | [Are bisphosphonates efficacious in knee osteoarthritis? A meta-analysis of randomized controlled trials.](https://www.ncbi.nlm.nih.gov/pubmed/29222056)  REJECT: medical treatment |
| --- | --- |
|  | Vaysbrot EE, Osani MC, Musetti MC, McAlindon TE, Bannuru RR. |
|  | Osteoarthritis Cartilage. 2018 Feb;26(2):154-164. doi: 10.1016/j.joca.2017.11.013. Epub 2017 Dec 6. |
|  | PMID: 29222056 [PubMed - indexed for MEDLINE]**Free Article** |
|  | [Similar articles](https://www.ncbi.nlm.nih.gov/pubmed?linkname=pubmed_pubmed&from_uid=29222056) |

| 18. | [Osteochondritis dissecans of the knee.](https://www.ncbi.nlm.nih.gov/pubmed/29197636)  REJECT: review |
| --- | --- |
|  | Accadbled F, Vial J, Sales de Gauzy J. |
|  | Orthop Traumatol Surg Res. 2018 Feb;104(1S):S97-S105. doi: 10.1016/j.otsr.2017.02.016. Epub 2017 Nov 29. Review. |
|  | PMID: 29197636 [PubMed - indexed for MEDLINE]**Free Article** |
|  | [Similar articles](https://www.ncbi.nlm.nih.gov/pubmed?linkname=pubmed_pubmed&from_uid=29197636) |

| 19. | [An in vivo model of a mechanically-induced bone marrow lesion.](https://www.ncbi.nlm.nih.gov/pubmed/29056291)  REJECT: posttraumatic osteoarthritis |
| --- | --- |
|  | Matheny JB, Goff MG, Pownder SL, Koff MF, Hayashi K, Yang X, Bostrom MPG, van der Meulen MCH, Hernandez CJ. |
|  | J Biomech. 2017 Nov 7;64:258-261. doi: 10.1016/j.jbiomech.2017.09.020. Epub 2017 Oct 13. |
|  | PMID: 29056291 [PubMed - indexed for MEDLINE]**Free PMC Article** |
|  | [Similar articles](https://www.ncbi.nlm.nih.gov/pubmed?linkname=pubmed_pubmed&from_uid=29056291) |

| 20. | [Arthritis Progression on Serial MRIs Following Diagnosis of Medial Meniscal Posterior Horn Root Tear.](https://www.ncbi.nlm.nih.gov/pubmed/28950387)  REJECT: posttraumatic osteoarthritis |
| --- | --- |
|  | Krych AJ, Johnson NR, Mohan R, Hevesi M, Stuart MJ, Littrell LA, Collins MS. |
|  | J Knee Surg. 2018 Aug;31(7):698-704. doi: 10.1055/s-0037-1607038. Epub 2017 Sep 26. Erratum in: [J Knee Surg. 2018 Aug;31(7):e1](https://www.ncbi.nlm.nih.gov/pubmed/29020692). |
|  | PMID: 28950387 [PubMed - indexed for MEDLINE] |
|  | [Similar articles](https://www.ncbi.nlm.nih.gov/pubmed?linkname=pubmed_pubmed&from_uid=28950387) |

| 21. | [Oral administration of undenatured native chicken type II collagen (UC-II) diminished deterioration of articular cartilage in a rat model of osteoarthritis (OA).](https://www.ncbi.nlm.nih.gov/pubmed/28888901)  REJECT: medical treatment |
| --- | --- |
|  | Bagi CM, Berryman ER, Teo S, Lane NE. |
|  | Osteoarthritis Cartilage. 2017 Dec;25(12):2080-2090. doi: 10.1016/j.joca.2017.08.013. Epub 2017 Sep 6. |
|  | PMID: 28888901 [PubMed - indexed for MEDLINE]**Free Article** |
|  | [Similar articles](https://www.ncbi.nlm.nih.gov/pubmed?linkname=pubmed_pubmed&from_uid=28888901) |

| 22. | [Joint loading and proximal tibia subchondral trabecular bone microarchitecture differ with walking gait patterns in end-stage knee osteoarthritis.](https://www.ncbi.nlm.nih.gov/pubmed/28642164)  REJECT: patient subgroup |
| --- | --- |
|  | Roberts BC, Solomon LB, Mercer G, Reynolds KJ, Thewlis D, Perilli E. |
|  | Osteoarthritis Cartilage. 2017 Oct;25(10):1623-1632. doi: 10.1016/j.joca.2017.06.001. Epub 2017 Jun 20. |
|  | PMID: 28642164 [PubMed - indexed for MEDLINE]**Free Article** |
|  | [Similar articles](https://www.ncbi.nlm.nih.gov/pubmed?linkname=pubmed_pubmed&from_uid=28642164) |

| 23. | [Comparison of Clinical and Radiological Parameters at Knee Osteoarthritis.](https://www.ncbi.nlm.nih.gov/pubmed/28428674) |
| --- | --- |
|  | Talic-Tanovic A, Hadziahmetovic Z, Madjar-Simic I, Papovic A. |
|  | Med Arch. 2017 Feb;71(1):48-51. doi: 10.5455/medarh.2017.71.48-51. Epub 2017 Feb 5. |
|  | PMID: 28428674 [PubMed - indexed for MEDLINE]**Free PMC Article** |
|  | [Similar articles](https://www.ncbi.nlm.nih.gov/pubmed?linkname=pubmed_pubmed&from_uid=28428674) |

| 24. | [Acromegalic arthropathy in various stages of the disease: an MRI study.](https://www.ncbi.nlm.nih.gov/pubmed/28348071)  REJECT: patient subgroup |
| --- | --- |
|  | Claessen KMJA, Canete AN, de Bruin PW, Pereira AM, Kloppenburg M, Kroon HM, Biermasz NR. |
|  | Eur J Endocrinol. 2017 Jun;176(6):779-790. doi: 10.1530/EJE-16-1073. Epub 2017 Mar 27. |
|  | PMID: 28348071 [PubMed - indexed for MEDLINE] |
|  | [Similar articles](https://www.ncbi.nlm.nih.gov/pubmed?linkname=pubmed_pubmed&from_uid=28348071) |

| 25. | [Effects of Treadmill Exercise on Advanced Osteoarthritis Pain in Rats.](https://www.ncbi.nlm.nih.gov/pubmed/28320059)  REJECT: interventional study |
| --- | --- |
|  | Allen J, Imbert I, Havelin J, Henderson T, Stevenson G, Liaw L, King T. |
|  | Arthritis Rheumatol. 2017 Jul;69(7):1407-1417. doi: 10.1002/art.40101. Epub 2017 Jun 5. |
|  | PMID: 28320059 [PubMed - indexed for MEDLINE]**Free PMC Article** |
|  | [Similar articles](https://www.ncbi.nlm.nih.gov/pubmed?linkname=pubmed_pubmed&from_uid=28320059) |

| 26. | [Perfusion of subchondral bone marrow in knee osteoarthritis: A dynamic contrast-enhanced magnetic resonance imaging preliminary study.](https://www.ncbi.nlm.nih.gov/pubmed/28189197) |
| --- | --- |
|  | Budzik JF, Ding J, Norberciak L, Pascart T, Toumi H, Verclytte S, Coursier R. |
|  | Eur J Radiol. 2017 Mar;88:129-134. doi: 10.1016/j.ejrad.2016.12.023. Epub 2016 Dec 24. |
|  | PMID: 28189197 [PubMed - indexed for MEDLINE] |
|  | [Similar articles](https://www.ncbi.nlm.nih.gov/pubmed?linkname=pubmed_pubmed&from_uid=28189197) |

| 27. | [High Bone Mass is associated with bone-forming features of osteoarthritis in non-weight bearing joints independent of body mass index.](https://www.ncbi.nlm.nih.gov/pubmed/28082078)  REJECT: study of bone density or mechanical properties of bone |
| --- | --- |
|  | Gregson CL, Hardcastle SA, Murphy A, Faber B, Fraser WD, Williams M, Davey Smith G, Tobias JH. |
|  | Bone. 2017 Apr;97:306-313. doi: 10.1016/j.bone.2017.01.005. Epub 2017 Jan 7. |
|  | PMID: 28082078 [PubMed - indexed for MEDLINE]**Free PMC Article** |
|  | [Similar articles](https://www.ncbi.nlm.nih.gov/pubmed?linkname=pubmed_pubmed&from_uid=28082078) |

| 28. | [Pulsed electromagnetic field at different stages of knee osteoarthritis in rats induced by low-dose monosodium iodoacetate: Effect on subchondral trabecular bone microarchitecture and cartilage degradation.](https://www.ncbi.nlm.nih.gov/pubmed/28026095)  REJECT: medical treatment |
| --- | --- |
|  | Yang X, He H, Zhou Y, Zhou Y, Gao Q, Wang P, He C. |
|  | Bioelectromagnetics. 2017 Apr;38(3):227-238. doi: 10.1002/bem.22028. Epub 2016 Dec 27. |
|  | PMID: 28026095 [PubMed - indexed for MEDLINE] |
|  | [Similar articles](https://www.ncbi.nlm.nih.gov/pubmed?linkname=pubmed_pubmed&from_uid=28026095) |

| 29. | [Thinning of articular cartilage after joint unloading or immobilization. An experimental investigation of the pathogenesis in mice.](https://www.ncbi.nlm.nih.gov/pubmed/27916560)  REJECT: interventional study |
| --- | --- |
|  | Nomura M, Sakitani N, Iwasawa H, Kohara Y, Takano S, Wakimoto Y, Kuroki H, Moriyama H. |
|  | Osteoarthritis Cartilage. 2017 May;25(5):727-736. doi: 10.1016/j.joca.2016.11.013. Epub 2016 Dec 1. |
|  | PMID: 27916560 [PubMed - indexed for MEDLINE]**Free Article** |
|  | [Similar articles](https://www.ncbi.nlm.nih.gov/pubmed?linkname=pubmed_pubmed&from_uid=27916560) |

| 30. | [Optimizing finite element predictions of local subchondral bone structural stiffness using neural network-derived density-modulus relationships for proximal tibial subchondral cortical and trabecular bone.](https://www.ncbi.nlm.nih.gov/pubmed/27842233)  REJECT: study of bone density or mechanical properties of bone |
| --- | --- |
|  | Nazemi SM, Amini M, Kontulainen SA, Milner JS, Holdsworth DW, Masri BA, Wilson DR, Johnston JD. |
|  | Clin Biomech (Bristol, Avon). 2017 Jan;41:1-8. doi: 10.1016/j.clinbiomech.2016.10.012. Epub 2016 Oct 27. |
|  | PMID: 27842233 [PubMed - indexed for MEDLINE] |
|  | [Similar articles](https://www.ncbi.nlm.nih.gov/pubmed?linkname=pubmed_pubmed&from_uid=27842233) |

| 31. | [Whole-body vibration of mice induces articular cartilage degeneration with minimal changes in subchondral bone.](https://www.ncbi.nlm.nih.gov/pubmed/27840128)  REJECT: interventional study |
| --- | --- |
|  | McCann MR, Yeung C, Pest MA, Ratneswaran A, Pollmann SI, Holdsworth DW, Beier F, Dixon SJ, Séguin CA. |
|  | Osteoarthritis Cartilage. 2017 May;25(5):770-778. doi: 10.1016/j.joca.2016.11.001. Epub 2016 Nov 10. |
|  | PMID: 27840128 [PubMed - indexed for MEDLINE]**Free Article** |
|  | [Similar articles](https://www.ncbi.nlm.nih.gov/pubmed?linkname=pubmed_pubmed&from_uid=27840128) |

| 32. | [Research in Osteochondritis Dissecans of the Knee: 2016 Update.](https://www.ncbi.nlm.nih.gov/pubmed/27532280)  REJECT: review |
| --- | --- |
|  | Nepple JJ, Milewski MD, Shea KG. |
|  | J Knee Surg. 2016 Oct;29(7):533-538. Epub 2016 Aug 17. Review. Erratum in: [J Knee Surg. 2016 Nov;29(8):696](https://www.ncbi.nlm.nih.gov/pubmed/27776368). |
|  | PMID: 27532280 [PubMed - indexed for MEDLINE] |
|  | [Similar articles](https://www.ncbi.nlm.nih.gov/pubmed?linkname=pubmed_pubmed&from_uid=27532280) |

| 33. | [Subchondral bone scan uptake correlates with articular cartilage degeneration in osteoarthritic knees.](https://www.ncbi.nlm.nih.gov/pubmed/27454909)  REJECT: study of bone density or mechanical properties of bone |
| --- | --- |
|  | Park DY, Jin LH, Min BH, Kwack KS, An YS, Kim YJ. |
|  | Int J Rheum Dis. 2017 Oct;20(10):1393-1402. doi: 10.1111/1756-185X.12909. Epub 2016 Jul 25. |
|  | PMID: 27454909 [PubMed - indexed for MEDLINE] |
|  | [Similar articles](https://www.ncbi.nlm.nih.gov/pubmed?linkname=pubmed_pubmed&from_uid=27454909) |

| 34. | [Autologous tissue transplantations for osteochondral repair.](https://www.ncbi.nlm.nih.gov/pubmed/27034191)  REJECT: review |
| --- | --- |
|  | Christensen BB. |
|  | Dan Med J. 2016 Apr;63(4). pii: B5236. Review. |
|  | PMID: 27034191 [PubMed - indexed for MEDLINE] |
|  | [Similar articles](https://www.ncbi.nlm.nih.gov/pubmed?linkname=pubmed_pubmed&from_uid=27034191) |

| 35. | [Three-Dimensional Quantitative Morphometric Analysis (QMA) for In Situ Joint and Tissue Assessment of Osteoarthritis in a Preclinical Rabbit Disease Model.](https://www.ncbi.nlm.nih.gov/pubmed/26808542)  REJECT: morphometric study |
| --- | --- |
|  | Stok KS, Besler BA, Steiner TH, Villarreal Escudero AV, Zulliger MA, Wilke M, Atal K, Quintin A, Koller B, Müller R, Nesic D. |
|  | PLoS One. 2016 Jan 25;11(1):e0147564. doi: 10.1371/journal.pone.0147564. eCollection 2016. |
|  | PMID: 26808542 [PubMed - indexed for MEDLINE]**Free PMC Article** |
|  | [Similar articles](https://www.ncbi.nlm.nih.gov/pubmed?linkname=pubmed_pubmed&from_uid=26808542) |

| 36. | [Computed tomography analysis of osteochondral defects of the talus after arthroscopic debridement and microfracture.](https://www.ncbi.nlm.nih.gov/pubmed/26713327)  REJECT: surgical procedure |
| --- | --- |
|  | Reilingh ML, van Bergen CJ, Blankevoort L, Gerards RM, van Eekeren IC, Kerkhoffs GM, van Dijk CN. |
|  | Knee Surg Sports Traumatol Arthrosc. 2016 Apr;24(4):1286-92. doi: 10.1007/s00167-015-3928-6. Epub 2015 Dec 28. |
|  | PMID: 26713327 [PubMed - indexed for MEDLINE]**Free PMC Article** |
|  | [Similar articles](https://www.ncbi.nlm.nih.gov/pubmed?linkname=pubmed_pubmed&from_uid=26713327) |

| 37. | [The relationships between bone mineral density and radiographic features of hand or knee osteoarthritis in older adults: data from the Dong-gu Study.](https://www.ncbi.nlm.nih.gov/pubmed/26467750)  REJECT: study of bone density or mechanical properties of bone |
| --- | --- |
|  | Wen L, Shin MH, Kang JH, Yim YR, Kim JE, Lee JW, Lee KE, Park DJ, Kim TJ, Park YW, Kweon SS, Lee YH, Yun YW, Lee SS. |
|  | Rheumatology (Oxford). 2016 Mar;55(3):495-503. doi: 10.1093/rheumatology/kev377. Epub 2015 Oct 13. |
|  | PMID: 26467750 [PubMed - indexed for MEDLINE] |
|  | [Similar articles](https://www.ncbi.nlm.nih.gov/pubmed?linkname=pubmed_pubmed&from_uid=26467750) |

| 38. | [Subchondral plate porosity colocalizes with the point of mechanical load during ambulation in a rat knee model of post-traumatic osteoarthritis.](https://www.ncbi.nlm.nih.gov/pubmed/26376125)  REJECT: posttraumatic osteoarthritis |
| --- | --- |
|  | Iijima H, Aoyama T, Tajino J, Ito A, Nagai M, Yamaguchi S, Zhang X, Kiyan W, Kuroki H. |
|  | Osteoarthritis Cartilage. 2016 Feb;24(2):354-63. doi: 10.1016/j.joca.2015.09.001. Epub 2015 Sep 14. |
|  | PMID: 26376125 [PubMed - indexed for MEDLINE]**Free Article** |
|  | [Similar articles](https://www.ncbi.nlm.nih.gov/pubmed?linkname=pubmed_pubmed&from_uid=26376125) |

| 39. | [Correlation between μCT imaging, histology and functional capacity of the osteoarthritic knee in the rat model of osteoarthritis.](https://www.ncbi.nlm.nih.gov/pubmed/26303725)  REJECT: histological study |
| --- | --- |
|  | Bagi CM, Zakur DE, Berryman E, Andresen CJ, Wilkie D. |
|  | J Transl Med. 2015 Aug 25;13:276. doi: 10.1186/s12967-015-0641-7. |
|  | PMID: 26303725 [PubMed - indexed for MEDLINE]**Free PMC Article** |
|  | [Similar articles](https://www.ncbi.nlm.nih.gov/pubmed?linkname=pubmed_pubmed&from_uid=26303725) |

| 40. | [Abnormal perfusion in patellofemoral subchondral bone marrow in the rat anterior cruciate ligament transection model of post-traumatic osteoarthritis: a dynamic contrast-enhanced magnetic resonance imaging study.](https://www.ncbi.nlm.nih.gov/pubmed/26241778)  REJECT: posttraumatic osteoarthritis |
| --- | --- |
|  | Tsai PH, Lee HS, Siow TY, Wang CY, Chang YC, Lin MH, Hsu YC, Lee CH, Chung HW, Huang GS. |
|  | Osteoarthritis Cartilage. 2016 Jan;24(1):129-33. doi: 10.1016/j.joca.2015.07.015. Epub 2015 Aug 1. |
|  | PMID: 26241778 [PubMed - indexed for MEDLINE]**Free Article** |
|  | [Similar articles](https://www.ncbi.nlm.nih.gov/pubmed?linkname=pubmed_pubmed&from_uid=26241778) |

| 41. | [Response to Letter to the Editor: 'Is subchondral bone mineral density associated with nocturnal pain in knee osteoarthritis patients?'.](https://www.ncbi.nlm.nih.gov/pubmed/26162805)  REJECT: letter |
| --- | --- |
|  | Burnett WD, Kontulainen SA, McLennan CE, Hazel D, Talmo C, Hunter DJ, Wilson DR, Johnston JD. |
|  | Osteoarthritis Cartilage. 2015 Dec;23(12):2299-2301. doi: 10.1016/j.joca.2015.06.015. Epub 2015 Jul 8. No abstract available. |
|  | PMID: 26162805 [PubMed - indexed for MEDLINE]**Free Article** |
|  | [Similar articles](https://www.ncbi.nlm.nih.gov/pubmed?linkname=pubmed_pubmed&from_uid=26162805) |

| 42. | [Is subchondral bone mineral density associated with nocturnal pain in knee osteoarthritis patients?](https://www.ncbi.nlm.nih.gov/pubmed/26162803)  REJECT: study of bone density or mechanical properties of bone |
| --- | --- |
|  | Chen Y, Huang YC, Lu WW. |
|  | Osteoarthritis Cartilage. 2015 Dec;23(12):2297-2298. doi: 10.1016/j.joca.2015.05.036. Epub 2015 Jul 8. No abstract available. |
|  | PMID: 26162803 [PubMed - indexed for MEDLINE]**Free Article** |
|  | [Similar articles](https://www.ncbi.nlm.nih.gov/pubmed?linkname=pubmed_pubmed&from_uid=26162803) |

| 43. | [Bone marrow lesions, subchondral bone cysts and subchondral bone attrition are associated with histological synovitis in patients with end-stage knee osteoarthritis: a cross-sectional study.](https://www.ncbi.nlm.nih.gov/pubmed/26028139)  REJECT: patient subgroup |
| --- | --- |
|  | Yusup A, Kaneko H, Liu L, Ning L, Sadatsuki R, Hada S, Kamagata K, Kinoshita M, Futami I, Shimura Y, Tsuchiya M, Saita Y, Takazawa Y, Ikeda H, Aoki S, Kaneko K, Ishijima M. |
|  | Osteoarthritis Cartilage. 2015 Nov;23(11):1858-64. doi: 10.1016/j.joca.2015.05.017. Epub 2015 May 29. |
|  | PMID: 26028139 [PubMed - indexed for MEDLINE]**Free Article** |
|  | [Similar articles](https://www.ncbi.nlm.nih.gov/pubmed?linkname=pubmed_pubmed&from_uid=26028139) |

| 44. | [Multi-scalar mechanical testing of the calcified cartilage and subchondral bone comparing healthy vs early degenerative states.](https://www.ncbi.nlm.nih.gov/pubmed/26028136)  REJECT: study of bone density or mechanical properties of cartilage |
| --- | --- |
|  | Hargrave-Thomas E, van Sloun F, Dickinson M, Broom N, Thambyah A. |
|  | Osteoarthritis Cartilage. 2015 Oct;23(10):1755-62. doi: 10.1016/j.joca.2015.05.012. Epub 2015 May 29. |
|  | PMID: 26028136 [PubMed - indexed for MEDLINE]**Free Article** |
|  | [Similar articles](https://www.ncbi.nlm.nih.gov/pubmed?linkname=pubmed_pubmed&from_uid=26028136) |

| 45. | [Prediction of local proximal tibial subchondral bone structural stiffness using subject-specific finite element modeling: Effect of selected density-modulus relationship.](https://www.ncbi.nlm.nih.gov/pubmed/26024555)  REJECT: study of bone density or mechanical properties of bone |
| --- | --- |
|  | Nazemi SM, Amini M, Kontulainen SA, Milner JS, Holdsworth DW, Masri BA, Wilson DR, Johnston JD. |
|  | Clin Biomech (Bristol, Avon). 2015 Aug;30(7):703-12. doi: 10.1016/j.clinbiomech.2015.05.002. Epub 2015 May 14. |
|  | PMID: 26024555 [PubMed - indexed for MEDLINE] |
|  | [Similar articles](https://www.ncbi.nlm.nih.gov/pubmed?linkname=pubmed_pubmed&from_uid=26024555) |

| 46. | [Effects of short-term gentle treadmill walking on subchondral bone in a rat model of instability-induced osteoarthritis.](https://www.ncbi.nlm.nih.gov/pubmed/25916553)  REJECT: interventional study |
| --- | --- |
|  | Iijima H, Aoyama T, Ito A, Yamaguchi S, Nagai M, Tajino J, Zhang X, Kuroki H. |
|  | Osteoarthritis Cartilage. 2015 Sep;23(9):1563-74. doi: 10.1016/j.joca.2015.04.015. Epub 2015 Apr 24. |
|  | PMID: 25916553 [PubMed - indexed for MEDLINE]**Free Article** |
|  | [Similar articles](https://www.ncbi.nlm.nih.gov/pubmed?linkname=pubmed_pubmed&from_uid=25916553) |

| 47. | [Knee osteoarthritis patients with severe nocturnal pain have altered proximal tibial subchondral bone mineral density.](https://www.ncbi.nlm.nih.gov/pubmed/25907860)  REJECT: study of bone density or mechanical properties of bone |
| --- | --- |
|  | Burnett WD, Kontulainen SA, McLennan CE, Hazel D, Talmo C, Hunter DJ, Wilson DR, Johnston JD. |
|  | Osteoarthritis Cartilage. 2015 Sep;23(9):1483-90. doi: 10.1016/j.joca.2015.04.012. Epub 2015 Apr 20. |
|  | PMID: 25907860 [PubMed - indexed for MEDLINE]**Free Article** |
|  | [Similar articles](https://www.ncbi.nlm.nih.gov/pubmed?linkname=pubmed_pubmed&from_uid=25907860) |

| 48. | [Intermittent applied mechanical loading induces subchondral bone thickening that may be intensified locally by contiguous articular cartilage lesions.](https://www.ncbi.nlm.nih.gov/pubmed/25655679)  REJECT: interventional study |
| --- | --- |
|  | Poulet B, de Souza R, Kent AV, Saxon L, Barker O, Wilson A, Chang YM, Cake M, Pitsillides AA. |
|  | Osteoarthritis Cartilage. 2015 Jun;23(6):940-8. doi: 10.1016/j.joca.2015.01.012. Epub 2015 Feb 2. |
|  | PMID: 25655679 [PubMed - indexed for MEDLINE]**Free PMC Article** |
|  | [Similar articles](https://www.ncbi.nlm.nih.gov/pubmed?linkname=pubmed_pubmed&from_uid=25655679) |

| 49. | [Subchondral insufficiency fractures of the knee: review of imaging findings.](https://www.ncbi.nlm.nih.gov/pubmed/24919465)  REJECT: review |
| --- | --- |
|  | Jose J, Pasquotti G, Smith MK, Gupta A, Lesniak BP, Kaplan LD. |
|  | Acta Radiol. 2015 Jun;56(6):714-9. doi: 10.1177/0284185114535132. Epub 2014 Jun 11. Review. |
|  | PMID: 24919465 [PubMed - indexed for MEDLINE] |
|  | [Similar articles](https://www.ncbi.nlm.nih.gov/pubmed?linkname=pubmed_pubmed&from_uid=24919465) |

| 50. | [Low magnitude high frequency vibration accelerated cartilage degeneration but improved epiphyseal bone formation in anterior cruciate ligament transect induced osteoarthritis rat model.](https://www.ncbi.nlm.nih.gov/pubmed/24852700)  REJECT: interventional study |
| --- | --- |
|  | Qin J, Chow SK, Guo A, Wong WN, Leung KS, Cheung WH. |
|  | Osteoarthritis Cartilage. 2014 Jul;22(7):1061-7. doi: 10.1016/j.joca.2014.05.004. Epub 2014 May 20. |
|  | PMID: 24852700 [PubMed - indexed for MEDLINE]**Free Article** |
|  | [Similar articles](https://www.ncbi.nlm.nih.gov/pubmed?linkname=pubmed_pubmed&from_uid=24852700) |

| 51. | [Correlation between 3D microstructural and 2D histomorphometric properties of subchondral bone with healthy and degenerative cartilage of the knee joint.](https://www.ncbi.nlm.nih.gov/pubmed/24828695)  REJECT: morphometric study |
| --- | --- |
|  | Lahm A, Kasch R, Spank H, Erggelet C, Esser J, Merk H, Mrosek E. |
|  | Histol Histopathol. 2014 Nov;29(11):1477-88. doi: 10.14670/HH-29.1477. Epub 2014 May 14. |
|  | PMID: 24828695 [PubMed - indexed for MEDLINE] |
|  | [Similar articles](https://www.ncbi.nlm.nih.gov/pubmed?linkname=pubmed_pubmed&from_uid=24828695) |

| 52. | [A comparison between platelet-rich plasma (PRP) and hyaluronate acid on the healing of cartilage defects.](https://www.ncbi.nlm.nih.gov/pubmed/24819118)  REJECT: medical treatment |
| --- | --- |
|  | Liu J, Song W, Yuan T, Xu Z, Jia W, Zhang C. |
|  | PLoS One. 2014 May 12;9(5):e97293. doi: 10.1371/journal.pone.0097293. eCollection 2014. |
|  | PMID: 24819118 [PubMed - indexed for MEDLINE]**Free PMC Article** |
|  | [Similar articles](https://www.ncbi.nlm.nih.gov/pubmed?linkname=pubmed_pubmed&from_uid=24819118) |

| 53. | [Cartilage repair procedures associated with high tibial osteotomy in varus knees: clinical results at 11 years' follow-up.](https://www.ncbi.nlm.nih.gov/pubmed/24507767)  REJECT: surgical procedure |
| --- | --- |
|  | Ferruzzi A, Buda R, Cavallo M, Timoncini A, Natali S, Giannini S. |
|  | Knee. 2014 Mar;21(2):445-50. doi: 10.1016/j.knee.2013.11.013. Epub 2013 Nov 27. |
|  | PMID: 24507767 [PubMed - indexed for MEDLINE] |
|  | [Similar articles](https://www.ncbi.nlm.nih.gov/pubmed?linkname=pubmed_pubmed&from_uid=24507767) |

| 54. | [Subchondral chitosan/blood implant-guided bone plate resorption and woven bone repair is coupled to hyaline cartilage regeneration from microdrill holes in aged rabbit knees.](https://www.ncbi.nlm.nih.gov/pubmed/24361795)  REJECT: interventional study |
| --- | --- |
|  | Guzmán-Morales J, Lafantaisie-Favreau CH, Chen G, Hoemann CD. |
|  | Osteoarthritis Cartilage. 2014 Feb;22(2):323-33. doi: 10.1016/j.joca.2013.12.011. Epub 2013 Dec 19. |
|  | PMID: 24361795 [PubMed - indexed for MEDLINE]**Free Article** |
|  | [Similar articles](https://www.ncbi.nlm.nih.gov/pubmed?linkname=pubmed_pubmed&from_uid=24361795) |

| 55. | [Deleterious effects of osteoarthritis on the structure and function of the meniscal enthesis.](https://www.ncbi.nlm.nih.gov/pubmed/24316288) |
| --- | --- |
|  | Abraham AC, Pauly HM, Donahue TL.  REJECT: histological study |
|  | Osteoarthritis Cartilage. 2014 Feb;22(2):275-83. doi: 10.1016/j.joca.2013.11.013. Epub 2013 Dec 5. |
|  | PMID: 24316288 [PubMed - indexed for MEDLINE]**Free PMC Article** |
|  | [Similar articles](https://www.ncbi.nlm.nih.gov/pubmed?linkname=pubmed_pubmed&from_uid=24316288) |

| 56. | [Regional depth-specific subchondral bone density measures in osteoarthritic and normal patellae: in vivo precision and preliminary comparisons.](https://www.ncbi.nlm.nih.gov/pubmed/24221452)  REJECT: study of bone density or mechanical properties of bone |
| --- | --- |
|  | Burnett WD, Kontulainen SA, McLennan CE, Hunter DJ, Wilson DR, Johnston JD. |
|  | Osteoporos Int. 2014 Mar;25(3):1107-14. doi: 10.1007/s00198-013-2568-2. Epub 2013 Nov 13. |
|  | PMID: 24221452 [PubMed - indexed for MEDLINE] |
|  | [Similar articles](https://www.ncbi.nlm.nih.gov/pubmed?linkname=pubmed_pubmed&from_uid=24221452) |

| 57. | [Efficacy of strength and aerobic exercise on patient-reported outcomes and structural changes in patients with knee osteoarthritis: study protocol for a randomized controlled trial.](https://www.ncbi.nlm.nih.gov/pubmed/24028201)  REJECT: interventional study |
| --- | --- |
|  | Øiestad BE, Østerås N, Frobell R, Grotle M, Brøgger H, Risberg MA. |
|  | BMC Musculoskelet Disord. 2013 Sep 12;14:266. doi: 10.1186/1471-2474-14-266. |
|  | PMID: 24028201 [PubMed - indexed for MEDLINE]**Free PMC Article** |
|  | [Similar articles](https://www.ncbi.nlm.nih.gov/pubmed?linkname=pubmed_pubmed&from_uid=24028201) |

| 58. | [Correlation between plasma, synovial fluid and articular cartilage Interleukin-18 with radiographic severity in 33 patients with osteoarthritis of the knee.](https://www.ncbi.nlm.nih.gov/pubmed/23958877)  REJECT: study of receptors, hormones or biological markers |
| --- | --- |
|  | Wang Y, Xu D, Long L, Deng X, Tao R, Huang G. |
|  | Clin Exp Med. 2014 Aug;14(3):297-304. |
|  | PMID: 23958877 [PubMed - indexed for MEDLINE] |
|  | [Similar articles](https://www.ncbi.nlm.nih.gov/pubmed?linkname=pubmed_pubmed&from_uid=23958877) |

| 59. | [Cross-sectional and longitudinal associations between systemic, subchondral bone mineral density and knee cartilage thickness in older adults with or without radiographic osteoarthritis.](https://www.ncbi.nlm.nih.gov/pubmed/23904471)  REJECT: study of bone density or mechanical properties of bone |
| --- | --- |
|  | Cao Y, Stannus OP, Aitken D, Cicuttini F, Antony B, Jones G, Ding C. |
|  | Ann Rheum Dis. 2014 Nov;73(11):2003-9. doi: 10.1136/annrheumdis-2013-203691. Epub 2013 Jul 31. |
|  | PMID: 23904471 [PubMed - indexed for MEDLINE] |
|  | [Similar articles](https://www.ncbi.nlm.nih.gov/pubmed?linkname=pubmed_pubmed&from_uid=23904471) |

| 60. | [Bone loss at subchondral plate in knee osteoarthritis patients with hypertension and type 2 diabetes mellitus.](https://www.ncbi.nlm.nih.gov/pubmed/23831668)  REJECT: patient subgroup |
| --- | --- |
|  | Wen CY, Chen Y, Tang HL, Yan CH, Lu WW, Chiu KY. |
|  | Osteoarthritis Cartilage. 2013 Nov;21(11):1716-23. doi: 10.1016/j.joca.2013.06.027. Epub 2013 Jul 4. |
|  | PMID: 23831668 [PubMed - indexed for MEDLINE]**Free Article** |
|  | [Similar articles](https://www.ncbi.nlm.nih.gov/pubmed?linkname=pubmed_pubmed&from_uid=23831668) |

| 61. | [The complexity of pain around the knee in patients with osteoarthritis.](https://www.ncbi.nlm.nih.gov/pubmed/23781753)  REJECT: review |
| --- | --- |
|  | Haviv B, Bronak S, Thein R. |
|  | Isr Med Assoc J. 2013 Apr;15(4):178-81. Review. |
|  | PMID: 23781753 [PubMed - indexed for MEDLINE]**Free Article** |
|  | [Similar articles](https://www.ncbi.nlm.nih.gov/pubmed?linkname=pubmed_pubmed&from_uid=23781753) |

| 62. | [Examination of osteoarthritis and subchondral bone alterations within the stifle joint of an ovariectomised ovine model.](https://www.ncbi.nlm.nih.gov/pubmed/23634692)  REJECT: interventional study |
| --- | --- |
|  | Holland JC, Brennan O, Kennedy OD, Mahony NJ, Rackard S, O'Brien FJ, Lee TC. |
|  | J Anat. 2013 Jun;222(6):588-97. doi: 10.1111/joa.12051. Epub 2013 May 2. |
|  | PMID: 23634692 [PubMed - indexed for MEDLINE]**Free PMC Article** |
|  | [Similar articles](https://www.ncbi.nlm.nih.gov/pubmed?linkname=pubmed_pubmed&from_uid=23634692) |

| 63. | [Spatial and temporal changes of subchondral bone proceed to microscopic articular cartilage degeneration in guinea pigs with spontaneous osteoarthritis.](https://www.ncbi.nlm.nih.gov/pubmed/23313833)  REJECT: histological study |
| --- | --- |
|  | Wang T, Wen CY, Yan CH, Lu WW, Chiu KY. |
|  | Osteoarthritis Cartilage. 2013 Apr;21(4):574-81. doi: 10.1016/j.joca.2013.01.002. Epub 2013 Jan 9. |
|  | PMID: 23313833 [PubMed - indexed for MEDLINE]**Free Article** |
|  | [Similar articles](https://www.ncbi.nlm.nih.gov/pubmed?linkname=pubmed_pubmed&from_uid=23313833) |

| 64. | [Clinical relevance of bone marrow lesions in OA.](https://www.ncbi.nlm.nih.gov/pubmed/23229449)  REJECT: review |
| --- | --- |
|  | Kwoh CK. |
|  | Nat Rev Rheumatol. 2013 Jan;9(1):7-8. doi: 10.1038/nrrheum.2012.217. Epub 2012 Dec 11. |
|  | PMID: 23229449 [PubMed - indexed for MEDLINE] |
|  | [Similar articles](https://www.ncbi.nlm.nih.gov/pubmed?linkname=pubmed_pubmed&from_uid=23229449) |

| 65. | [CT imaging for the investigation of subchondral bone in knee osteoarthritis.](https://www.ncbi.nlm.nih.gov/pubmed/23179574)  REJECT: review |
| --- | --- |
|  | Bousson V, Lowitz T, Laouisset L, Engelke K, Laredo JD. |
|  | Osteoporos Int. 2012 Dec;23 Suppl 8:S861-5. doi: 10.1007/s00198-012-2169-5. Epub 2012 Nov 22. Review. |
|  | PMID: 23179574 [PubMed - indexed for MEDLINE] |
|  | [Similar articles](https://www.ncbi.nlm.nih.gov/pubmed?linkname=pubmed_pubmed&from_uid=23179574) |

| 66. | [Assessment of bone mineral density and radiographic texture analysis at the tibial subchondral bone.](https://www.ncbi.nlm.nih.gov/pubmed/23179572)  REJECT: review |
| --- | --- |
|  | Lespessailles E, Jennane R. |
|  | Osteoporos Int. 2012 Dec;23 Suppl 8:S871-6. doi: 10.1007/s00198-012-2167-7. Epub 2012 Nov 22. Review. |
|  | PMID: 23179572 [PubMed - indexed for MEDLINE] |
|  | [Similar articles](https://www.ncbi.nlm.nih.gov/pubmed?linkname=pubmed_pubmed&from_uid=23179572) |

| 67. | [Aromatase inhibitors associated with knee subchondral bone expansion without cartilage loss.](https://www.ncbi.nlm.nih.gov/pubmed/23148546)  REJECT: interventional study |
| --- | --- |
|  | Davis SR, Bell RJ, Wang Y, Hanna F, Davies-Tuck M, Bell R, Chirgwin J, Cicuttini F. |
|  | Climacteric. 2013 Dec;16(6):632-8. doi: 10.3109/13697137.2012.746656. Epub 2012 Dec 10. |
|  | PMID: 23148546 [PubMed - indexed for MEDLINE] |
|  | [Similar articles](https://www.ncbi.nlm.nih.gov/pubmed?linkname=pubmed_pubmed&from_uid=23148546) |

| 68. | [Bone and cartilage demonstrate changes localized to bone marrow edema-like lesions within osteoarthritic knees.](https://www.ncbi.nlm.nih.gov/pubmed/23025926)  REJECT: test of new imaging technique |
| --- | --- |
|  | Kazakia GJ, Kuo D, Schooler J, Siddiqui S, Shanbhag S, Bernstein G, Horvai A, Majumdar S, Ries M, Li X. |
|  | Osteoarthritis Cartilage. 2013 Jan;21(1):94-101. doi: 10.1016/j.joca.2012.09.008. Epub 2012 Sep 28. |
|  | PMID: 23025926 [PubMed - indexed for MEDLINE]**Free PMC Article** |
|  | [Similar articles](https://www.ncbi.nlm.nih.gov/pubmed?linkname=pubmed_pubmed&from_uid=23025926) |

| 69. | [[Knee joint osteoarthrosis: risk factors of rapid progression].](https://www.ncbi.nlm.nih.gov/pubmed/22830211)  REJECT: non-English language |
| --- | --- |
|  | [No authors listed] |
|  | Ter Arkh. 2012;84(5):42-5. Russian. |
|  | PMID: 22830211 [PubMed - indexed for MEDLINE] |
|  | [Similar articles](https://www.ncbi.nlm.nih.gov/pubmed?linkname=pubmed_pubmed&from_uid=22830211) |

| 70. | [Associations between pre-operative radiographic changes and outcomes after total knee joint replacement for osteoarthritis.](https://www.ncbi.nlm.nih.gov/pubmed/22800770)  REJECT: surgical procedure |
| --- | --- |
|  | Dowsey MM, Nikpour M, Dieppe P, Choong PF. |
|  | Osteoarthritis Cartilage. 2012 Oct;20(10):1095-102. doi: 10.1016/j.joca.2012.05.015. Epub 2012 Jul 16. |
|  | PMID: 22800770 [PubMed - indexed for MEDLINE]**Free Article** |
|  | [Similar articles](https://www.ncbi.nlm.nih.gov/pubmed?linkname=pubmed_pubmed&from_uid=22800770) |

| 71. | [Magnetic resonance imaging of subchondral bone marrow lesions in association with osteoarthritis.](https://www.ncbi.nlm.nih.gov/pubmed/22542276)  REJECT: review |
| --- | --- |
|  | Xu L, Hayashi D, Roemer FW, Felson DT, Guermazi A. |
|  | Semin Arthritis Rheum. 2012 Oct;42(2):105-18. doi: 10.1016/j.semarthrit.2012.03.009. Epub 2012 Apr 26. Review. |
|  | PMID: 22542276 [PubMed - indexed for MEDLINE]**Free PMC Article** |
|  | [Similar articles](https://www.ncbi.nlm.nih.gov/pubmed?linkname=pubmed_pubmed&from_uid=22542276) |

| 72. | [Low bone mineral density is associated with the onset of spontaneous osteonecrosis of the knee.](https://www.ncbi.nlm.nih.gov/pubmed/22537352) |
| --- | --- |
|  | Akamatsu Y, Mitsugi N, Hayashi T, Kobayashi H, Saito T.  REJECT: study of bone density or mechanical properties of bone |
|  | Acta Orthop. 2012 Jun;83(3):249-55. doi: 10.3109/17453674.2012.684139. Epub 2012 Apr 27. |
|  | PMID: 22537352 [PubMed - indexed for MEDLINE]**Free PMC Article** |
|  | [Similar articles](https://www.ncbi.nlm.nih.gov/pubmed?linkname=pubmed_pubmed&from_uid=22537352) |

| 73. | [Altered trabecular bone structure and delayed cartilage degeneration in the knees of collagen VI null mice.](https://www.ncbi.nlm.nih.gov/pubmed/22448243)  REJECT: animal subgroup |
| --- | --- |
|  | Christensen SE, Coles JM, Zelenski NA, Furman BD, Leddy HA, Zauscher S, Bonaldo P, Guilak F. |
|  | PLoS One. 2012;7(3):e33397. doi: 10.1371/journal.pone.0033397. Epub 2012 Mar 20. |
|  | PMID: 22448243 [PubMed - indexed for MEDLINE]**Free PMC Article** |
|  | [Similar articles](https://www.ncbi.nlm.nih.gov/pubmed?linkname=pubmed_pubmed&from_uid=22448243) |

| 74. | [Cartilage and bone changes during development of post-traumatic osteoarthritis in selected LGXSM recombinant inbred mice.](https://www.ncbi.nlm.nih.gov/pubmed/22361237)  REJECT: posttraumatic osteoarthritis model |
| --- | --- |
|  | Hashimoto S, Rai MF, Janiszak KL, Cheverud JM, Sandell LJ. |
|  | Osteoarthritis Cartilage. 2012 Jun;20(6):562-71. doi: 10.1016/j.joca.2012.01.022. Epub 2012 Feb 8. |
|  | PMID: 22361237 [PubMed - indexed for MEDLINE]**Free PMC Article** |
|  | [Similar articles](https://www.ncbi.nlm.nih.gov/pubmed?linkname=pubmed_pubmed&from_uid=22361237) |

| 75. | [The role of calcified cartilage and subchondral bone in the initiation and progression of ochronotic arthropathy in alkaptonuria.](https://www.ncbi.nlm.nih.gov/pubmed/22127706)  REJECT: patient subgroup |
| --- | --- |
|  | Taylor AM, Boyde A, Wilson PJ, Jarvis JC, Davidson JS, Hunt JA, Ranganath LR, Gallagher JA. |
|  | Arthritis Rheum. 2011 Dec;63(12):3887-96. doi: 10.1002/art.30606. |
|  | PMID: 22127706 [PubMed - indexed for MEDLINE]**Free Article** |
|  | [Similar articles](https://www.ncbi.nlm.nih.gov/pubmed?linkname=pubmed_pubmed&from_uid=22127706) |

| 76. | [Malalignment and subchondral bone turnover in contralateral knees of overweight/obese women with unilateral osteoarthritis: implications for bilateral disease.](https://www.ncbi.nlm.nih.gov/pubmed/22034115) |
| --- | --- |
|  | Mazzuca SA, Brandt KD, Lane KA, Chakr R. |
|  | Arthritis Care Res (Hoboken). 2011 Nov;63(11):1528-34. doi: 10.1002/acr.20574. |
|  | PMID: 22034115 [PubMed - indexed for MEDLINE]**Free Article** |
|  | [Similar articles](https://www.ncbi.nlm.nih.gov/pubmed?linkname=pubmed_pubmed&from_uid=22034115) |

| 77. | [Arthrodiatasis for management of knee osteoarthritis.](https://www.ncbi.nlm.nih.gov/pubmed/21815573)  REJECT: surgical procedure |
| --- | --- |
|  | Aly TA, Hafez K, Amin O. |
|  | Orthopedics. 2011 Aug 8;34(8):e338-43. doi: 10.3928/01477447-20110627-17. |
|  | PMID: 21815573 [PubMed - indexed for MEDLINE] |
|  | [Similar articles](https://www.ncbi.nlm.nih.gov/pubmed?linkname=pubmed_pubmed&from_uid=21815573) |

| 78. | [Grape seed proanthocyanidin extract ameliorates monosodium iodoacetate-induced osteoarthritis.](https://www.ncbi.nlm.nih.gov/pubmed/21795829)  REJECT: medical treatment |
| --- | --- |
|  | Woo YJ, Joo YB, Jung YO, Ju JH, Cho ML, Oh HJ, Jhun JY, Park MK, Park JS, Kang CM, Sung MS, Park SH, Kim HY, Min JK. |
|  | Exp Mol Med. 2011 Oct 31;43(10):561-70. doi: 10.3858/emm.2011.43.10.062. |
|  | PMID: 21795829 [PubMed - indexed for MEDLINE]**Free PMC Article** |
|  | [Similar articles](https://www.ncbi.nlm.nih.gov/pubmed?linkname=pubmed_pubmed&from_uid=21795829) |

| 79. | [Changes in articular cartilage mechanics with meniscectomy: A novel image-based modeling approach and comparison to patterns of OA.](https://www.ncbi.nlm.nih.gov/pubmed/21741046)  REJECT: interventional study |
| --- | --- |
|  | Haemer JM, Song Y, Carter DR, Giori NJ. |
|  | J Biomech. 2011 Aug 11;44(12):2307-12. doi: 10.1016/j.jbiomech.2011.04.014. Epub 2011 Jul 7. |
|  | PMID: 21741046 [PubMed - indexed for MEDLINE] |
|  | [Similar articles](https://www.ncbi.nlm.nih.gov/pubmed?linkname=pubmed_pubmed&from_uid=21741046) |

| 80. | [Associations of anatomical measures from MRI with radiographically defined knee osteoarthritis score, pain, and physical functioning.](https://www.ncbi.nlm.nih.gov/pubmed/21266638) |
| --- | --- |
|  | Sowers M, Karvonen-Gutierrez CA, Jacobson JA, Jiang Y, Yosef M. |
|  | J Bone Joint Surg Am. 2011 Feb 2;93(3):241-51. doi: 10.2106/JBJS.I.00667. |
|  | PMID: 21266638 [PubMed - indexed for MEDLINE]**Free PMC Article** |
|  | [Similar articles](https://www.ncbi.nlm.nih.gov/pubmed?linkname=pubmed_pubmed&from_uid=21266638) |

| 81. | [Biochemical markers in the diagnosis of chondral defects following anterior cruciate ligament insufficiency.](https://www.ncbi.nlm.nih.gov/pubmed/21221577)  REJECT: patient subgroup |
| --- | --- |
|  | Streich NA, Zimmermann D, Schmitt H, Bode G. |
|  | Int Orthop. 2011 Nov;35(11):1633-7. doi: 10.1007/s00264-010-1191-5. Epub 2011 Jan 11. |
|  | PMID: 21221577 [PubMed - indexed for MEDLINE]**Free PMC Article** |
|  | [Similar articles](https://www.ncbi.nlm.nih.gov/pubmed?linkname=pubmed_pubmed&from_uid=21221577) |

| 82. | [2-year postoperative evaluation of a patient with a symptomatic full-thickness patellar cartilage defect repaired with particulated juvenile cartilage tissue.](https://www.ncbi.nlm.nih.gov/pubmed/21141688)  REJECT: surgical procedure |
| --- | --- |
|  | Bonner KF, Daner W, Yao JQ. |
|  | J Knee Surg. 2010 Jun;23(2):109-14. |
|  | PMID: 21141688 [PubMed - indexed for MEDLINE] |
|  | [Similar articles](https://www.ncbi.nlm.nih.gov/pubmed?linkname=pubmed_pubmed&from_uid=21141688) |

| 83. | [Analysis of osteoarthritis in a mouse model of the progeroid human DNA repair syndrome trichothiodystrophy.](https://www.ncbi.nlm.nih.gov/pubmed/20820927)  REJECT: patient subgroup |
| --- | --- |
|  | Botter SM, Zar M, van Osch GJ, van Steeg H, Dollé ME, Hoeijmakers JH, Weinans H, van Leeuwen JP. |
|  | Age (Dordr). 2011 Sep;33(3):247-60. doi: 10.1007/s11357-010-9175-3. Epub 2010 Sep 7. |
|  | PMID: 20820927 [PubMed - indexed for MEDLINE]**Free PMC Article** |
|  | [Similar articles](https://www.ncbi.nlm.nih.gov/pubmed?linkname=pubmed_pubmed&from_uid=20820927) |

| 84. | [Magnetic resonance imaging evaluation of weight-bearing subchondral trabecular bone in the knee.](https://www.ncbi.nlm.nih.gov/pubmed/20449585)  REJECT: test of new imaging technique |
| --- | --- |
|  | Schneider E, Lo GH, Sloane G, Fanella L, Hunter DJ, Eaton CB, McAlindon TE. |
|  | Skeletal Radiol. 2011 Jan;40(1):95-103. doi: 10.1007/s00256-010-0943-z. Epub 2010 May 7. |
|  | PMID: 20449585 [PubMed - indexed for MEDLINE]**Free PMC Article** |
|  | [Similar articles](https://www.ncbi.nlm.nih.gov/pubmed?linkname=pubmed_pubmed&from_uid=20449585) |

| 85. | [Assessment of the radii of the medial and lateral femoral condyles in varus and valgus knees with osteoarthritis.](https://www.ncbi.nlm.nih.gov/pubmed/20048101)  REJECT: surgical procedure |
| --- | --- |
|  | Howell SM, Howell SJ, Hull ML. |
|  | J Bone Joint Surg Am. 2010 Jan;92(1):98-104. doi: 10.2106/JBJS.H.01566. |
|  | PMID: 20048101 [PubMed - indexed for MEDLINE] |
|  | [Similar articles](https://www.ncbi.nlm.nih.gov/pubmed?linkname=pubmed_pubmed&from_uid=20048101) |

| 86. | [Denuded subchondral bone and knee pain in persons with knee osteoarthritis.](https://www.ncbi.nlm.nih.gov/pubmed/19950284) |
| --- | --- |
|  | Moisio K, Eckstein F, Chmiel JS, Guermazi A, Prasad P, Almagor O, Song J, Dunlop D, Hudelmaier M, Kothari A, Sharma L. |
|  | Arthritis Rheum. 2009 Dec;60(12):3703-10. doi: 10.1002/art.25014. |
|  | PMID: 19950284 [PubMed - indexed for MEDLINE]**Free PMC Article** |
|  | [Similar articles](https://www.ncbi.nlm.nih.gov/pubmed?linkname=pubmed_pubmed&from_uid=19950284) |

| 87. | [Trabecular morphometry by fractal signature analysis is a novel marker of osteoarthritis progression.](https://www.ncbi.nlm.nih.gov/pubmed/19950282)  REJECT: test of new imaging technique |
| --- | --- |
|  | Kraus VB, Feng S, Wang S, White S, Ainslie M, Brett A, Holmes A, Charles HC. |
|  | Arthritis Rheum. 2009 Dec;60(12):3711-22. doi: 10.1002/art.25012. |
|  | PMID: 19950282 [PubMed - indexed for MEDLINE]**Free PMC Article** |
|  | [Similar articles](https://www.ncbi.nlm.nih.gov/pubmed?linkname=pubmed_pubmed&from_uid=19950282) |

| 88. | [Imaging insights on the epidemiology and pathophysiology of osteoarthritis.](https://www.ncbi.nlm.nih.gov/pubmed/19931798)  REJECT: review |
| --- | --- |
|  | Hunter DJ. |
|  | Rheum Dis Clin North Am. 2009 Aug;35(3):447-63. doi: 10.1016/j.rdc.2009.08.001. Review. |
|  | PMID: 19931798 [PubMed - indexed for MEDLINE] |
|  | [Similar articles](https://www.ncbi.nlm.nih.gov/pubmed?linkname=pubmed_pubmed&from_uid=19931798) |

| 89. | [Pre-radiographic MRI findings are associated with onset of knee symptoms: the most study.](https://www.ncbi.nlm.nih.gov/pubmed/19919856) |
| --- | --- |
|  | Javaid MK, Lynch JA, Tolstykh I, Guermazi A, Roemer F, Aliabadi P, McCulloch C, Curtis J, Felson D, Lane NE, Torner J, Nevitt M. |
|  | Osteoarthritis Cartilage. 2010 Mar;18(3):323-8. doi: 10.1016/j.joca.2009.11.002. Epub 2009 Nov 11. |
|  | PMID: 19919856 [PubMed - indexed for MEDLINE]**Free PMC Article** |
|  | [Similar articles](https://www.ncbi.nlm.nih.gov/pubmed?linkname=pubmed_pubmed&from_uid=19919856) |

| 90. | [Subchondral bone attrition may be a reflection of compartment-specific mechanical load: the MOST Study.](https://www.ncbi.nlm.nih.gov/pubmed/19762366) |
| --- | --- |
|  | Neogi T, Nevitt M, Niu J, Sharma L, Roemer F, Guermazi A, Lewis CE, Torner J, Javaid K, Felson D. |
|  | Ann Rheum Dis. 2010 May;69(5):841-4. doi: 10.1136/ard.2009.110114. Epub 2009 Sep 17. |
|  | PMID: 19762366 [PubMed - indexed for MEDLINE]**Free PMC Article** |
|  | [Similar articles](https://www.ncbi.nlm.nih.gov/pubmed?linkname=pubmed_pubmed&from_uid=19762366) |

| 91. | [Patellar tendon versus hamstring tendon autografts for anterior cruciate ligament reconstruction: a randomized controlled trial using similar femoral and tibial fixation methods.](https://www.ncbi.nlm.nih.gov/pubmed/19684298)  REJECT: surgical procedure |
| --- | --- |
|  | Taylor DC, DeBerardino TM, Nelson BJ, Duffey M, Tenuta J, Stoneman PD, Sturdivant RX, Mountcastle S. |
|  | Am J Sports Med. 2009 Oct;37(10):1946-57. doi: 10.1177/0363546509339577. Epub 2009 Aug 14. |
|  | PMID: 19684298 [PubMed - indexed for MEDLINE] |
|  | [Similar articles](https://www.ncbi.nlm.nih.gov/pubmed?linkname=pubmed_pubmed&from_uid=19684298) |

| 92. | [Insights from imaging on the epidemiology and pathophysiology of osteoarthritis.](https://www.ncbi.nlm.nih.gov/pubmed/19631067)  REJECT: review |
| --- | --- |
|  | Hunter DJ. |
|  | Radiol Clin North Am. 2009 Jul;47(4):539-51. doi: 10.1016/j.rcl.2009.03.004. Review. |
|  | PMID: 19631067 [PubMed - indexed for MEDLINE] |
|  | [Similar articles](https://www.ncbi.nlm.nih.gov/pubmed?linkname=pubmed_pubmed&from_uid=19631067) |

| 93. | [Subchondral fluid dynamics in a model of osteoarthritis: use of dynamic contrast-enhanced magnetic resonance imaging.](https://www.ncbi.nlm.nih.gov/pubmed/19409292) |
| --- | --- |
|  | Lee JH, Dyke JP, Ballon D, Ciombor DM, Rosenwasser MP, Aaron RK. |
|  | Osteoarthritis Cartilage. 2009 Oct;17(10):1350-5. doi: 10.1016/j.joca.2009.03.019. Epub 2009 Apr 17. |
|  | PMID: 19409292 [PubMed - indexed for MEDLINE]**Free PMC Article** |
|  | [Similar articles](https://www.ncbi.nlm.nih.gov/pubmed?linkname=pubmed_pubmed&from_uid=19409292) |

| 94. | [[Causes of pain in the knee joint].](https://www.ncbi.nlm.nih.gov/pubmed/18819352)  REJECT: non-English |
| --- | --- |
|  | Men'shikova IV, Babyre VV. |
|  | Klin Med (Mosk). 2008;86(8):67-70. Russian. |
|  | PMID: 18819352 [PubMed - indexed for MEDLINE] |
|  | [Similar articles](https://www.ncbi.nlm.nih.gov/pubmed?linkname=pubmed_pubmed&from_uid=18819352) |

| 95. | [A new non-invasive method to assess synovitis severity in relation to symptoms and cartilage volume loss in knee osteoarthritis patients using MRI.](https://www.ncbi.nlm.nih.gov/pubmed/18672386)  REJECT: test of new imaging technique |
| --- | --- |
|  | Pelletier JP, Raynauld JP, Abram F, Haraoui B, Choquette D, Martel-Pelletier J. |
|  | Osteoarthritis Cartilage. 2008;16 Suppl 3:S8-13. doi: 10.1016/j.joca.2008.06.007. Epub 2008 Jul 30. |
|  | PMID: 18672386 [PubMed - indexed for MEDLINE]**Free Article** |
|  | [Similar articles](https://www.ncbi.nlm.nih.gov/pubmed?linkname=pubmed_pubmed&from_uid=18672386) |

| 96. | [A pilot study of the reproducibility and validity of measuring knee subchondral bone density in the tibia.](https://www.ncbi.nlm.nih.gov/pubmed/18515160)  REJECT: test of new imaging technique |
| --- | --- |
|  | Dore D, Ding C, Jones G. |
|  | Osteoarthritis Cartilage. 2008 Dec;16(12):1539-44. doi: 10.1016/j.joca.2008.04.012. Epub 2008 Jun 2. |
|  | PMID: 18515160 [PubMed - indexed for MEDLINE]**Free Article** |
|  | [Similar articles](https://www.ncbi.nlm.nih.gov/pubmed?linkname=pubmed_pubmed&from_uid=18515160) |

| 97. | [Meniscectomy alters the dynamic deformational behavior and cumulative strain of tibial articular cartilage in knee joints subjected to cyclic loads.](https://www.ncbi.nlm.nih.gov/pubmed/18514552)  REJECT: surgical procedure |
| --- | --- |
|  | Song Y, Greve JM, Carter DR, Giori NJ. |
|  | Osteoarthritis Cartilage. 2008 Dec;16(12):1545-54. doi: 10.1016/j.joca.2008.04.011. Epub 2008 Jun 2. |
|  | PMID: 18514552 [PubMed - indexed for MEDLINE]**Free Article** |
|  | [Similar articles](https://www.ncbi.nlm.nih.gov/pubmed?linkname=pubmed_pubmed&from_uid=18514552) |

| 98. | [A type I collagen defect leads to rapidly progressive osteoarthritis in a mouse model.](https://www.ncbi.nlm.nih.gov/pubmed/18383364)  REJECT: patient or animal subgroup |
| --- | --- |
|  | Blair-Levy JM, Watts CE, Fiorentino NM, Dimitriadis EK, Marini JC, Lipsky PE. |
|  | Arthritis Rheum. 2008 Apr;58(4):1096-106. doi: 10.1002/art.23277. Erratum in: Arthritis Rheum. 2008 Jun;58(6):1888. Fiorientino, N M [corrected to Fiorentino, N M]. |
|  | PMID: 18383364 [PubMed - indexed for MEDLINE]**Free Article** |
|  | [Similar articles](https://www.ncbi.nlm.nih.gov/pubmed?linkname=pubmed_pubmed&from_uid=18383364) |

| 99. | [Meniscal damage associated with increased local subchondral bone mineral density: a Framingham study.](https://www.ncbi.nlm.nih.gov/pubmed/17825586)  REJECT: study of bone density or mechanical properties of bone |
| --- | --- |
|  | Lo GH, Niu J, McLennan CE, Kiel DP, McLean RR, Guermazi A, Genant HK, McAlindon TE, Hunter DJ. |
|  | Osteoarthritis Cartilage. 2008 Feb;16(2):261-7. Epub 2007 Sep 7. |
|  | PMID: 17825586 [PubMed - indexed for MEDLINE]**Free PMC Article** |
|  | [Similar articles](https://www.ncbi.nlm.nih.gov/pubmed?linkname=pubmed_pubmed&from_uid=17825586) |

| 100. | [Correlation between bone lesion changes and cartilage volume loss in patients with osteoarthritis of the knee as assessed by quantitative magnetic resonance imaging over a 24-month period.](https://www.ncbi.nlm.nih.gov/pubmed/17728333) |
| --- | --- |
|  | Raynauld JP, Martel-Pelletier J, Berthiaume MJ, Abram F, Choquette D, Haraoui B, Beary JF, Cline GA, Meyer JM, Pelletier JP. |
|  | Ann Rheum Dis. 2008 May;67(5):683-8. Epub 2007 Aug 29. |
|  | PMID: 17728333 [PubMed - indexed for MEDLINE] |
|  | [Similar articles](https://www.ncbi.nlm.nih.gov/pubmed?linkname=pubmed_pubmed&from_uid=17728333) |

| 101. | [Risk factors associated with the loss of cartilage volume on weight-bearing areas in knee osteoarthritis patients assessed by quantitative magnetic resonance imaging: a longitudinal study.](https://www.ncbi.nlm.nih.gov/pubmed/17672891) |
| --- | --- |
|  | Pelletier JP, Raynauld JP, Berthiaume MJ, Abram F, Choquette D, Haraoui B, Beary JF, Cline GA, Meyer JM, Martel-Pelletier J. |
|  | Arthritis Res Ther. 2007;9(4):R74. |
|  | PMID: 17672891 [PubMed - indexed for MEDLINE]**Free PMC Article** |
|  | [Similar articles](https://www.ncbi.nlm.nih.gov/pubmed?linkname=pubmed_pubmed&from_uid=17672891) |

| 102. | [Physical activity and knee structural change: a longitudinal study using MRI.](https://www.ncbi.nlm.nih.gov/pubmed/17473768) |
| --- | --- |
|  | Foley S, Ding C, Cicuttini F, Jones G. |
|  | Med Sci Sports Exerc. 2007 Mar;39(3):426-34. |
|  | PMID: 17473768 [PubMed - indexed for MEDLINE] |
|  | [Similar articles](https://www.ncbi.nlm.nih.gov/pubmed?linkname=pubmed_pubmed&from_uid=17473768) |

| 103. | [Association of bone marrow lesions with knee structures and risk factors for bone marrow lesions in the knees of clinically healthy, community-based adults.](https://www.ncbi.nlm.nih.gov/pubmed/17391738) |
| --- | --- |
|  | Baranyay FJ, Wang Y, Wluka AE, English DR, Giles GG, Sullivan RO, Cicuttini FM. |
|  | Semin Arthritis Rheum. 2007 Oct;37(2):112-8. Epub 2007 Mar 27. |
|  | PMID: 17391738 [PubMed - indexed for MEDLINE] |
|  | [Similar articles](https://www.ncbi.nlm.nih.gov/pubmed?linkname=pubmed_pubmed&from_uid=17391738) |

| 104. | [Knee meniscal extrusion in a largely non-osteoarthritic cohort: association with greater loss of cartilage volume.](https://www.ncbi.nlm.nih.gov/pubmed/17359552) |
| --- | --- |
|  | Ding C, Martel-Pelletier J, Pelletier JP, Abram F, Raynauld JP, Cicuttini F, Jones G. |
|  | Arthritis Res Ther. 2007;9(2):R21. |
|  | PMID: 17359552 [PubMed - indexed for MEDLINE]**Free PMC Article** |
|  | [Similar articles](https://www.ncbi.nlm.nih.gov/pubmed?linkname=pubmed_pubmed&from_uid=17359552) |

| 105. | [Influence of high tibial osteotomy on bone marrow edema in the knee.](https://www.ncbi.nlm.nih.gov/pubmed/16936577)  REJECT: surgical procedure |
| --- | --- |
|  | Kröner AH, Berger CE, Kluger R, Oberhauser G, Bock P, Engel A. |
|  | Clin Orthop Relat Res. 2007 Jan;454:155-62. |
|  | PMID: 16936577 [PubMed - indexed for MEDLINE] |
|  | [Similar articles](https://www.ncbi.nlm.nih.gov/pubmed?linkname=pubmed_pubmed&from_uid=16936577) |

| 106. | [A 2 yr longitudinal radiographic study examining the effect of a bisphosphonate (risedronate) upon subchondral bone loss in osteoarthritic knee patients.](https://www.ncbi.nlm.nih.gov/pubmed/16837470)  REJECT: medical treatment |
| --- | --- |
|  | Buckland-Wright JC, Messent EA, Bingham CO 3rd, Ward RJ, Tonkin C. |
|  | Rheumatology (Oxford). 2007 Feb;46(2):257-64. Epub 2006 Jul 11. |
|  | PMID: 16837470 [PubMed - indexed for MEDLINE] |
|  | [Similar articles](https://www.ncbi.nlm.nih.gov/pubmed?linkname=pubmed_pubmed&from_uid=16837470) |

| 107. | [Bone mineral density in the proximal tibia varies as a function of static alignment and knee adduction angular momentum in individuals with medial knee osteoarthritis.](https://www.ncbi.nlm.nih.gov/pubmed/16782419)  REJECT: study of bone density or mechanical properties of bone |
| --- | --- |
|  | Thorp LE, Wimmer MA, Block JA, Moisio KC, Shott S, Goker B, Sumner DR. |
|  | Bone. 2006 Nov;39(5):1116-1122. doi: 10.1016/j.bone.2006.05.001. Epub 2006 Jun 16. |
|  | PMID: 16782419 [PubMed - indexed for MEDLINE] |
|  | [Similar articles](https://www.ncbi.nlm.nih.gov/pubmed?linkname=pubmed_pubmed&from_uid=16782419) |

| 108. | [Osteoarthritis of the knee: association between clinical features and MR imaging findings.](https://www.ncbi.nlm.nih.gov/pubmed/16714463) |
| --- | --- |
|  | Kornaat PR, Bloem JL, Ceulemans RY, Riyazi N, Rosendaal FR, Nelissen RG, Carter WO, Hellio Le Graverand MP, Kloppenburg M. |
|  | Radiology. 2006 Jun;239(3):811-7. |
|  | PMID: 16714463 [PubMed - indexed for MEDLINE] |
|  | [Similar articles](https://www.ncbi.nlm.nih.gov/pubmed?linkname=pubmed_pubmed&from_uid=16714463) |

| 109. | [Associations between pain, function, and radiographic features in osteoarthritis of the knee.](https://www.ncbi.nlm.nih.gov/pubmed/16385522) |
| --- | --- |
|  | Szebenyi B, Hollander AP, Dieppe P, Quilty B, Duddy J, Clarke S, Kirwan JR. |
|  | Arthritis Rheum. 2006 Jan;54(1):230-5. |
|  | PMID: 16385522 [PubMed - indexed for MEDLINE]**Free Article** |
|  | [Similar articles](https://www.ncbi.nlm.nih.gov/pubmed?linkname=pubmed_pubmed&from_uid=16385522) |

| 110. | [Quantitative ultrasound imaging of spontaneous repair of porcine cartilage.](https://www.ncbi.nlm.nih.gov/pubmed/16288971)  REJECT: interventional study |
| --- | --- |
|  | Laasanen MS, Töyräs J, Vasara A, Saarakkala S, Hyttinen MM, Kiviranta I, Jurvelin JS. |
|  | Osteoarthritis Cartilage. 2006 Mar;14(3):258-63. Epub 2005 Nov 9. |
|  | PMID: 16288971 [PubMed - indexed for MEDLINE]**Free Article** |
|  | [Similar articles](https://www.ncbi.nlm.nih.gov/pubmed?linkname=pubmed_pubmed&from_uid=16288971) |

| 111. | [Macroscopic and microscopic features of synovial membrane inflammation in the osteoarthritic knee: correlating magnetic resonance imaging findings with disease severity.](https://www.ncbi.nlm.nih.gov/pubmed/16255041) |
| --- | --- |
|  | Loeuille D, Chary-Valckenaere I, Champigneulle J, Rat AC, Toussaint F, Pinzano-Watrin A, Goebel JC, Mainard D, Blum A, Pourel J, Netter P, Gillet P. |
|  | Arthritis Rheum. 2005 Nov;52(11):3492-501. |
|  | PMID: 16255041 [PubMed - indexed for MEDLINE]**Free Article** |
|  | [Similar articles](https://www.ncbi.nlm.nih.gov/pubmed?linkname=pubmed_pubmed&from_uid=16255041) |

| 112. | [Assessing bone loss on radiographs of the knee in osteoarthritis: a cross-sectional study.](https://www.ncbi.nlm.nih.gov/pubmed/16255025) |
| --- | --- |
|  | Dieppe PA, Reichenbach S, Williams S, Gregg P, Watt I, Jüni P. |
|  | Arthritis Rheum. 2005 Nov;52(11):3536-41. |
|  | PMID: 16255025 [PubMed - indexed for MEDLINE]**Free Article** |
|  | [Similar articles](https://www.ncbi.nlm.nih.gov/pubmed?linkname=pubmed_pubmed&from_uid=16255025) |

| 113. | [Bone marrow abnormalities on magnetic resonance imaging are associated with type II collagen degradation in knee osteoarthritis: a three-month longitudinal study.](https://www.ncbi.nlm.nih.gov/pubmed/16145678)  REJECT: study of receptors, hormones or biological markers |
| --- | --- |
|  | Garnero P, Peterfy C, Zaim S, Schoenharting M. |
|  | Arthritis Rheum. 2005 Sep;52(9):2822-9. |
|  | PMID: 16145678 [PubMed - indexed for MEDLINE]**Free Article** |
|  | [Similar articles](https://www.ncbi.nlm.nih.gov/pubmed?linkname=pubmed_pubmed&from_uid=16145678) |

| 114. | [Effects of hyaluronan on three-dimensional microarchitecture of subchondral bone tissues in guinea pig primary osteoarthrosis.](https://www.ncbi.nlm.nih.gov/pubmed/15777671)  REJECT: medical treatment |
| --- | --- |
|  | Ding M, Christian Danielsen C, Hvid I. |
|  | Bone. 2005 Mar;36(3):489-501. |
|  | PMID: 15777671 [PubMed - indexed for MEDLINE] |
|  | [Similar articles](https://www.ncbi.nlm.nih.gov/pubmed?linkname=pubmed_pubmed&from_uid=15777671) |

| 115. | [Long-term periarticular bone adaptation in a feline knee injury model for post-traumatic experimental osteoarthritis.](https://www.ncbi.nlm.nih.gov/pubmed/15727890)  REJECT: posttraumatic osteoarthritis |
| --- | --- |
|  | Boyd SK, Müller R, Leonard T, Herzog W. |
|  | Osteoarthritis Cartilage. 2005 Mar;13(3):235-42. |
|  | PMID: 15727890 [PubMed - indexed for MEDLINE]**Free Article** |
|  | [Similar articles](https://www.ncbi.nlm.nih.gov/pubmed?linkname=pubmed_pubmed&from_uid=15727890) |

| 116. | [Structural associations of osteoarthritis pain: lessons from magnetic resonance imaging.](https://www.ncbi.nlm.nih.gov/pubmed/15283451)  REJECT: review |
| --- | --- |
|  | Conaghan PG, Felson DT. |
|  | Novartis Found Symp. 2004;260:191-201; discussion 201-5, 277-9. Review. |
|  | PMID: 15283451 [PubMed - indexed for MEDLINE] |
|  | [Similar articles](https://www.ncbi.nlm.nih.gov/pubmed?linkname=pubmed_pubmed&from_uid=15283451) |

| 117. | [The fate of chondrocyte in osteoarthritic cartilage of transgenic mice expressing bovine GH.](https://www.ncbi.nlm.nih.gov/pubmed/15219569)  REJECT: study of receptors, hormones or biological markers |
| --- | --- |
|  | Fernández-Criado C, Martos-Rodríguez A, Santos-Alvarez I, García-Ruíz JP, Delgado-Baeza E. |
|  | Osteoarthritis Cartilage. 2004 Jul;12(7):543-51. |
|  | PMID: 15219569 [PubMed - indexed for MEDLINE]**Free Article** |
|  | [Similar articles](https://www.ncbi.nlm.nih.gov/pubmed?linkname=pubmed_pubmed&from_uid=15219569) |

| 118. | [The natural history of spontaneous osteonecrosis of the medial tibial plateau.](https://www.ncbi.nlm.nih.gov/pubmed/14516032) |
| --- | --- |
|  | Satku K, Kumar VP, Chong SM, Thambyah A. |
|  | J Bone Joint Surg Br. 2003 Sep;85(7):983-8. |
|  | PMID: 14516032 [PubMed - indexed for MEDLINE] |
|  | [Similar articles](https://www.ncbi.nlm.nih.gov/pubmed?linkname=pubmed_pubmed&from_uid=14516032) |

| 119. | [Material properties of subchondral bone from patients with osteoporosis or osteoarthritis by microindentation testing and electron probe microanalysis.](https://www.ncbi.nlm.nih.gov/pubmed/14506956)  REJECT: study of bone density or mechanical properties of bone |
| --- | --- |
|  | Coats AM, Zioupos P, Aspden RM. |
|  | Calcif Tissue Int. 2003 Jul;73(1):66-71. |
|  | PMID: 14506956 [PubMed - indexed for MEDLINE] |
|  | [Similar articles](https://www.ncbi.nlm.nih.gov/pubmed?linkname=pubmed_pubmed&from_uid=14506956) |

| 120. | [Mechanical and architectural bone adaptation in early stage experimental osteoarthritis.](https://www.ncbi.nlm.nih.gov/pubmed/11918226)  REJECT: interventional study |
| --- | --- |
|  | Boyd SK, Müller R, Zernicke RF. |
|  | J Bone Miner Res. 2002 Apr;17(4):687-94. |
|  | PMID: 11918226 [PubMed - indexed for MEDLINE]**Free Article** |
|  | [Similar articles](https://www.ncbi.nlm.nih.gov/pubmed?linkname=pubmed_pubmed&from_uid=11918226) |

| 121. | [Bone density of the human talus does not increase with the cartilage degeneration score.](https://www.ncbi.nlm.nih.gov/pubmed/11788940)  REJECT: study of bone density or mechanical properties of bone |
| --- | --- |
|  | Muehleman C, Berzins A, Koepp H, Eger W, Cole AA, Kuettner KE, Sumner DR. |
|  | Anat Rec. 2002 Feb 1;266(2):81-6. |
|  | PMID: 11788940 [PubMed - indexed for MEDLINE]**Free Article** |
|  | [Similar articles](https://www.ncbi.nlm.nih.gov/pubmed?linkname=pubmed_pubmed&from_uid=11788940) |

| 122. | [Contribution of arthroscopy and magnetic resonance imaging to the evaluation of painful knee osteoarthritis.](https://www.ncbi.nlm.nih.gov/pubmed/11195312)  REJECT: review |
| --- | --- |
|  | Boyer T, Daumen-Legré V. |
|  | Joint Bone Spine. 2000;67(6):504-8. Review. |
|  | PMID: 11195312 [PubMed - indexed for MEDLINE] |
|  | [Similar articles](https://www.ncbi.nlm.nih.gov/pubmed?linkname=pubmed_pubmed&from_uid=11195312) |

| 123. | [Evidence of early subchondral bone changes in the meniscectomized guinea pig. A densitometric study using dual-energy X-ray absorptiometry subregional analysis.](https://www.ncbi.nlm.nih.gov/pubmed/10489319)  REJECT: study of bone density or mechanical properties of bone |
| --- | --- |
|  | Pastoureau PC, Chomel AC, Bonnet J. |
|  | Osteoarthritis Cartilage. 1999 Sep;7(5):466-73. |
|  | PMID: 10489319 [PubMed - indexed for MEDLINE]**Free Article** |
|  | [Similar articles](https://www.ncbi.nlm.nih.gov/pubmed?linkname=pubmed_pubmed&from_uid=10489319) |

| 124. | [[Relationship between knee pain and X-ray findings of osteoarthritis with reference to bone mineral density measured by computed X-ray densitometry].](https://www.ncbi.nlm.nih.gov/pubmed/9396322)  REJECT: non-English |
| --- | --- |
|  | Miyamura T, Kinoshita T, Asaka A. |
|  | Nihon Ronen Igakkai Zasshi. 1997 Aug;34(8):657-61. Japanese. |
|  | PMID: 9396322 [PubMed - indexed for MEDLINE]**Free Article** |
|  | [Similar articles](https://www.ncbi.nlm.nih.gov/pubmed?linkname=pubmed_pubmed&from_uid=9396322) |

| 125. | [Osteoarthritis in cynomolgus macaques. III: Effects of age, gender, and subchondral bone thickness on the severity of disease.](https://www.ncbi.nlm.nih.gov/pubmed/8864894)  REJECT: animal subgroup |
| --- | --- |
|  | Carlson CS, Loeser RF, Purser CB, Gardin JF, Jerome CP. |
|  | J Bone Miner Res. 1996 Sep;11(9):1209-17. |
|  | PMID: 8864894 [PubMed - indexed for MEDLINE] |
|  | [Similar articles](https://www.ncbi.nlm.nih.gov/pubmed?linkname=pubmed_pubmed&from_uid=8864894) |

| 126. | [Relationship between serum cartilage oligomeric matrix protein levels and disease progression in osteoarthritis of the knee joint.](https://www.ncbi.nlm.nih.gov/pubmed/7788143)  REJECT: study of receptors, hormones or biological markers |
| --- | --- |
|  | Sharif M, Saxne T, Shepstone L, Kirwan JR, Elson CJ, Heinegård D, Dieppe PA. |
|  | Br J Rheumatol. 1995 Apr;34(4):306-10. |
|  | PMID: 7788143 [PubMed - indexed for MEDLINE] |
|  | [Similar articles](https://www.ncbi.nlm.nih.gov/pubmed?linkname=pubmed_pubmed&from_uid=7788143) |

| 127. | [Bone mineral distribution of the proximal tibia in gonarthrosis assessed in vivo by photon absorption.](https://www.ncbi.nlm.nih.gov/pubmed/11548230)  REJECT: study of bone density or mechanical properties of bone |
| --- | --- |
|  | Madsen OR, Schaadt O, Bliddal H, Egsmose C, Sylvest J. |
|  | Osteoarthritis Cartilage. 1994 Jun;2(2):141-7. |
|  | PMID: 11548230 [PubMed – indexed for MEDLINE] |
|  | [Similar articles](https://www.ncbi.nlm.nih.gov/pubmed?linkname=pubmed_pubmed&from_uid=11548230) |

| 128. | [Morphology-mechanical property relations in trabecular bone of the osteoarthritic proximal tibia.](https://www.ncbi.nlm.nih.gov/pubmed/8014652) |
| --- | --- |
|  | REJECT: study of bone density or mechanical properties of bone |
|  | Zysset PK, Sonny M, Hayes WC. |
|  | J Arthroplasty. 1994 Apr;9(2):203-16. |
|  | PMID: 8014652 [PubMed - indexed for MEDLINE] |
|  | [Similar articles](https://www.ncbi.nlm.nih.gov/pubmed?linkname=pubmed_pubmed&from_uid=8014652) |

| 129. | [[Spontaneous course of femorotibial osteoarthritis].](https://www.ncbi.nlm.nih.gov/pubmed/8239456)  REJECT: non-English |
| --- | --- |
|  | Glimet T. |
|  | Ann Radiol (Paris). 1993;36(3):220-4. French. |
|  | PMID: 8239456 [PubMed - indexed for MEDLINE] |
|  | [Similar articles](https://www.ncbi.nlm.nih.gov/pubmed?linkname=pubmed_pubmed&from_uid=8239456) |

| 130. | [Grading system of articular cartilage degeneration in osteoarthritis of the knee.](https://www.ncbi.nlm.nih.gov/pubmed/8012267)  REJECT: surgical study |
| --- | --- |
|  | Koshino T, Machida J. |
|  | Bull Hosp Jt Dis. 1993 Summer;53(3):41-6. |
|  | PMID: 8012267 [PubMed - indexed for MEDLINE] |
|  | [Similar articles](https://www.ncbi.nlm.nih.gov/pubmed?linkname=pubmed_pubmed&from_uid=8012267) |

| 131. | [Scintigraphic assessment of osteoarthritis of the knee joint.](https://www.ncbi.nlm.nih.gov/pubmed/1417117)  REJECT: test of new imaging technique |
| --- | --- |
|  | McCrae F, Shouls J, Dieppe P, Watt I. |
|  | Ann Rheum Dis. 1992 Aug;51(8):938-42. |
|  | PMID: 1417117 [PubMed - indexed for MEDLINE]**Free PMC Article** |
|  | [Similar articles](https://www.ncbi.nlm.nih.gov/pubmed?linkname=pubmed_pubmed&from_uid=1417117) |

| 132. | [Bone and cartilage changes following experimental varus or valgus tibial angulation.](https://www.ncbi.nlm.nih.gov/pubmed/2355297)  REJECT: interventional study |
| --- | --- |
|  | Wu DD, Burr DB, Boyd RD, Radin EL. |
|  | J Orthop Res. 1990 Jul;8(4):572-85. |
|  | PMID: 2355297 [PubMed - indexed for MEDLINE] |
|  | [Similar articles](https://www.ncbi.nlm.nih.gov/pubmed?linkname=pubmed_pubmed&from_uid=2355297) |

| 133. | [[Subchondral bone wear of the tibial plateau in femorotibial knee osteoarthritis. Radiologic aspects in the profile incidence. Clinical, anatomical correlations and consequences].](https://www.ncbi.nlm.nih.gov/pubmed/2320928)  REJECT: non-English |
| --- | --- |
|  | Hernigou P, Goutallier D. |
|  | Rev Rhum Mal Osteoartic. 1990 Jan;57(1):67-72. French. No abstract available. |
|  | PMID: 2320928 [PubMed - indexed for MEDLINE] |
|  | [Similar articles](https://www.ncbi.nlm.nih.gov/pubmed?linkname=pubmed_pubmed&from_uid=2320928) |

| 134. | [Autogenous bone grafting for severe angular deformity in total knee arthroplasty.](https://www.ncbi.nlm.nih.gov/pubmed/2664086)  REJECT: surgical procedure |
| --- | --- |
|  | Altchek D, Sculco TP, Rawlins B. |
|  | J Arthroplasty. 1989;4(2):151-5. |
|  | PMID: 2664086 [PubMed - indexed for MEDLINE] |
|  | [Similar articles](https://www.ncbi.nlm.nih.gov/pubmed?linkname=pubmed_pubmed&from_uid=2664086) |

| 135. | [[Mechanical behavior of the subchondral bone in the experimentally induced osteoarthritis].](https://www.ncbi.nlm.nih.gov/pubmed/490015)  REJECT: non-English |
| --- | --- |
|  | Miyanaga Y. |
|  | Nihon Seikeigeka Gakkai Zasshi. 1979 Jun;53(6):681-95. Japanese. |
|  | PMID: 490015 [PubMed - indexed for MEDLINE] |
|  | [Similar articles](https://www.ncbi.nlm.nih.gov/pubmed?linkname=pubmed_pubmed&from_uid=490015) |
